# Current controversies on mechanisms controlling soil carbon storage: implications for interactions with practitioners and policy-makers. A review

**DOI:** 10.1007/s13593-023-00876-x

**Published:** 2023-02-06

**Authors:** Delphine Derrien, Pierre Barré, Isabelle Basile-Doelsch, Lauric Cécillon, Abad Chabbi, Alexandra Crème, Sébastien Fontaine, Ludovic Henneron, Noémie Janot, Gwenaëlle Lashermes, Katell Quénéa, Frédéric Rees, Marie-France Dignac

**Affiliations:** 1 INRAE, BEF, F-54000 Nancy, France; 2 Laboratoire de Géologie, École Normale Supérieure, CNRS, PSL University, IPSL, Paris, France; 3 Aix Marseille University, CNRS, IRD, INRAE, Coll France, CEREGE, Aix-en-Provence, France; 4 UMR EcoSys, INRAE, AgroParisTech, Université Paris-Saclay, 78850 Thiverval-Grignon, France; 5 Université Clermont Auvergne, INRAE, VetAgro Sup, UMR Ecosystème Prairial, 63000 Clermont-Ferrand, France; 6 USC ECODIV-Rouen 7603, Normandie Université, UNIROUEN, INRAE, 76000 Rouen, France; 7 ISPA, Bordeaux Sciences Agro, INRAE, F-33140 Villenave d’Ornon, France; 8 Université de Reims Champagne Ardenne, INRAE, FARE, UMR A 614, 51097 Reims, France; 9 Sorbonne Université, CNRS, EPHE, PSL, UMR METIS, F-75005 Paris, France; 10 INRAE, CNRS, Sorbonne Université, UMR iEES-Paris, 4 place Jussieu, 75005 Paris, France

**Keywords:** Carbon storage, Chemical recalcitrance, POM, Inaccessibility, Models, Big data, Biomass use, Management practices

## Abstract

There is currently an intense debate about the potential for additional organic carbon storage in soil, the strategies by which it may be accomplished and what the actual benefits might be for agriculture and the climate. Controversy forms an essential part of the scientific process, but on the topic of soil carbon storage, it may confuse the agricultural community and the general public and may delay actions to fight climate change. In an attempt to shed light on this topic, the originality of this article lies in its intention to provide a balanced description of contradictory scientific opinions on soil carbon storage and to examine how the scientific community can support decision-making despite the controversy. In the first part, we review and attempt to reconcile conflicting views on the mechanisms controlling organic carbon dynamics in soil. We discuss the divergent opinions about chemical recalcitrance, the microbial or plant origin of persistent soil organic matter, the contribution of particulate organic matter to additional organic carbon storage in soil, and the spatial and energetic inaccessibility of soil organic matter to decomposers. In the second part, we examine the advantages and limitations of big data management and modeling, which are essential tools to link the latest scientific theories with the actions taken by stakeholders. Finally, we show how the analysis and discussion of controversies can guide scientists in supporting stakeholders for the design of (i) appropriate trade-offs for biomass use in agriculture and forestry and (ii) climate-smart management practices, keeping in mind their still unresolved effects on soil carbon storage.

## Contents

- 1. Introduction
- 2. Controversial issues and new challenges in research on mechanisms controlling C storage in soils
  - 2.1 Chemical recalcitrance: Should it be rehabilitated?
    - 2.1.1 A concept partly based on questionable methods
    - 2.1.2 A concept partly based on misunderstood results
    - 2.1.3 Revisiting recalcitrance in 142 light of other persistence processes
    - 2.1.4 How can recalcitrance be rehabilitated, and how can it be used in a practical way?
  - 2.2 Microbial transformation of plant organic matter: Is it a prerequisite for its persistence in soils?
    - 2.2.1 How microbial compounds are preserved in soil
    - 2.2.2 How plant compounds are preserved in soil
  - 2.3 Is particulate organic matter an effective lever for climate change mitigation?
    - 2.3.1 Properties of POM
    - 2.3.2 Contribution of POM to soil C stock
    - 2.3.3 Residence time of C in the POM fraction
    - 2.3.4 Limits and benefits of POM for C storage in soil
  - 2.4 Is soil organic matter persistence driven by spatial or energetic inaccessibility?
    - 2.4.1 Spatial inaccessibility
    - 2.4.2 Energetic inaccessibility
    - 2.4.3 The energetic dimension of spatial inaccessibility
    - 2.4.4 Nutrient issues, a third mechanism of inaccessibility limiting soil OM decomposition
  - 2.5 Reconciling competing theories of C persistence
- 3. Integrating mechanisms into databases and models
  - 3.1 Big data: opportunity or danger for research in soil science?
    - 3.1.1 Opportunities of big data
    - 3.1.2 Points of caution associated with big data
    - 3.1.3 Roadmap for the deployment of big data in soil science
  - 3.2 Is it necessary to integrate novel mechanistic knowledge in models of SOM dynamics?
    - 3.2.1 Benefits and limits of 631 process-based models
    - 3.2.2 Toward models adapted to users' expectations
  - 3.3 Databases and models: summary of benefits and pitfalls
- 4. Debates on the effects of harvest and management practices: how can research support practitioner and policy-maker decisions?
  - 4.1 How can acceptable trade-offs be designed regarding the use of plant biomass?
  - 4.2 Priorities for research on SOM mechanisms to support the implementation of practices beneficial to C accumulation in soil
- 5. Conclusions and perspectives
- Acknowledgements
- References

## Introduction

Throughout history, controversies have played a critical role in advancing scientific progress. They are essential to the emergence, development, and critical evaluation of concepts and methods. Disagreement can also stimulate new ways of interpreting data and help integrate different viewpoints on a subject (scientific, economic, ethical, political) (Dunlop and Veneu 2019). Although this process is essential to the advancement of research, scientific controversies are sometimes misinterpreted by the agricultural community and the general public and may undermine support for the scientific community. Scientific controversies also hamper the provision of univocal scientific knowledge to inform decision-making.

The recent example of the COVID-19 crisis has illustrated the difficulty of taking rapid action in the context of scientific controversy. In a similar way, ongoing debates about the practices and mechanisms controlling the storage of carbon (C) in soil run the risk of impeding climate change mitigation strategies. In particular, the “4 per 1000” initiative, which aims to promote actions and practices that can store C in soil—with associated benefits for food security and climate (Soussana et al. 2019)—has generated intense debates (Minasny et al. 2017 and the subsequent related commentary papers). The controversies associated with 4 per 1000 are both technical and political. Technical considerations relate to the methods used to calculate soil C stocks, possible overestimations, quantification of the effects of biomass uses, issues related to nutrient cycling or greenhouse gas emissions, and to the definition of an initial baseline (Larrère 2018). On the political side, the initiative could be used as a pretext for not reducing anthropogenic C emissions.

**Fig. 1:**
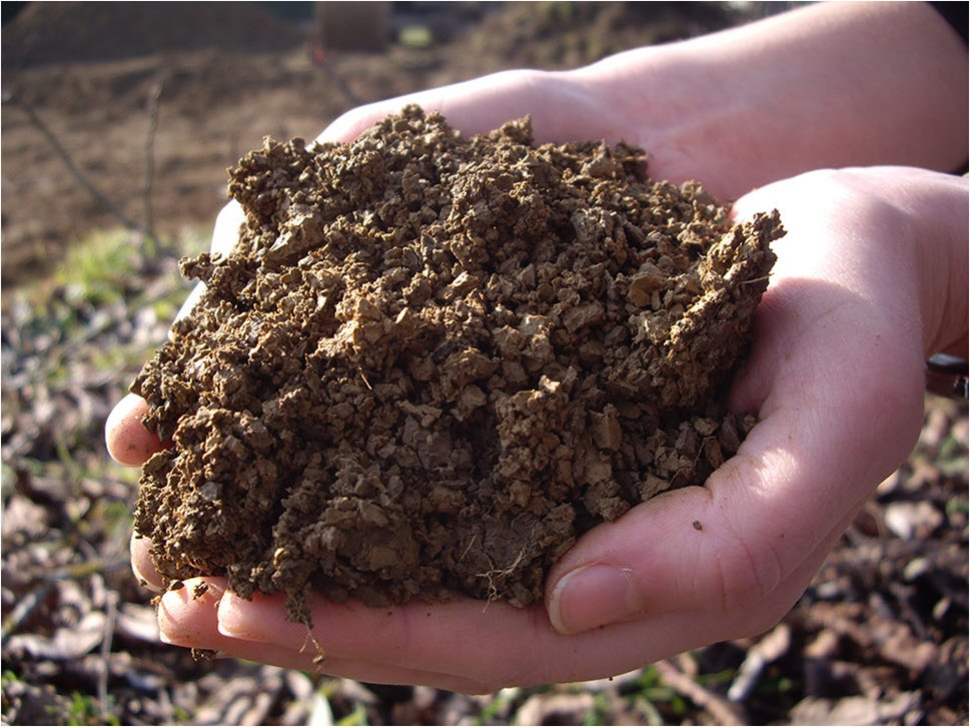
The future of soil C sequestration is in our hands (credit: D. Derrien).

**Fig. 2:**
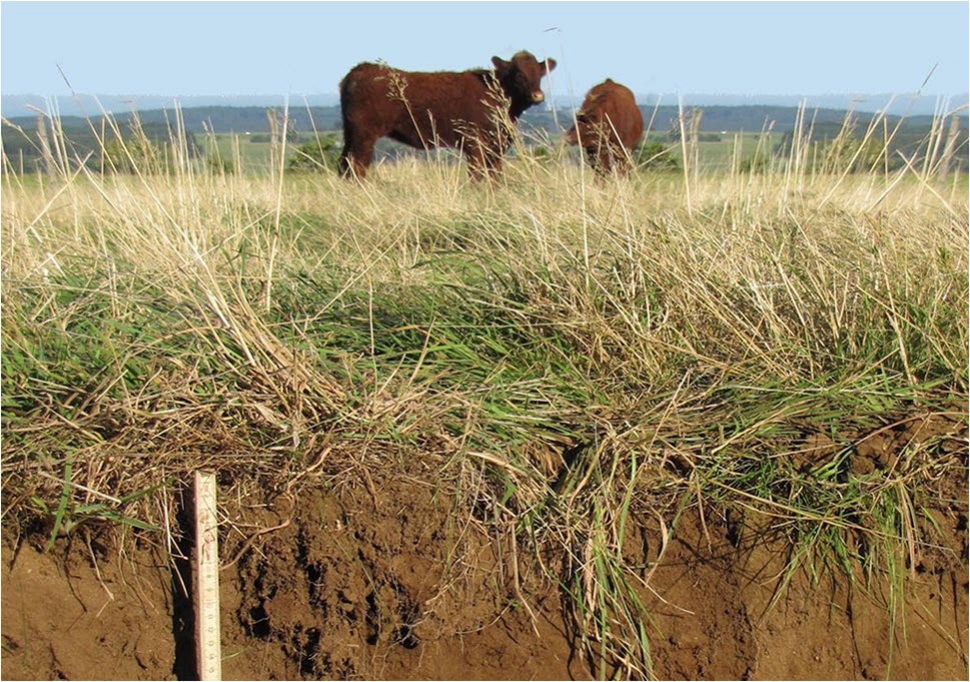
Everything we do on the soil has an impact on the soil (credit: J. Balesdent).

Debating some of the current technical controversies related to the “4 per 1000” initiative was the objective of the second seminar of the scientific network CarboSMS (Carbon Stabilization Mechanisms in Soil), which serves as the inspiration for this review. The CarboSMS collective brings together both researchers and stakeholders from the French-speaking community who work with scientific and operational issues related to the mechanisms that affect C storage in soil (Dignac et al. 2017) (Fig. 1 and 2).

**Fig. 3:**
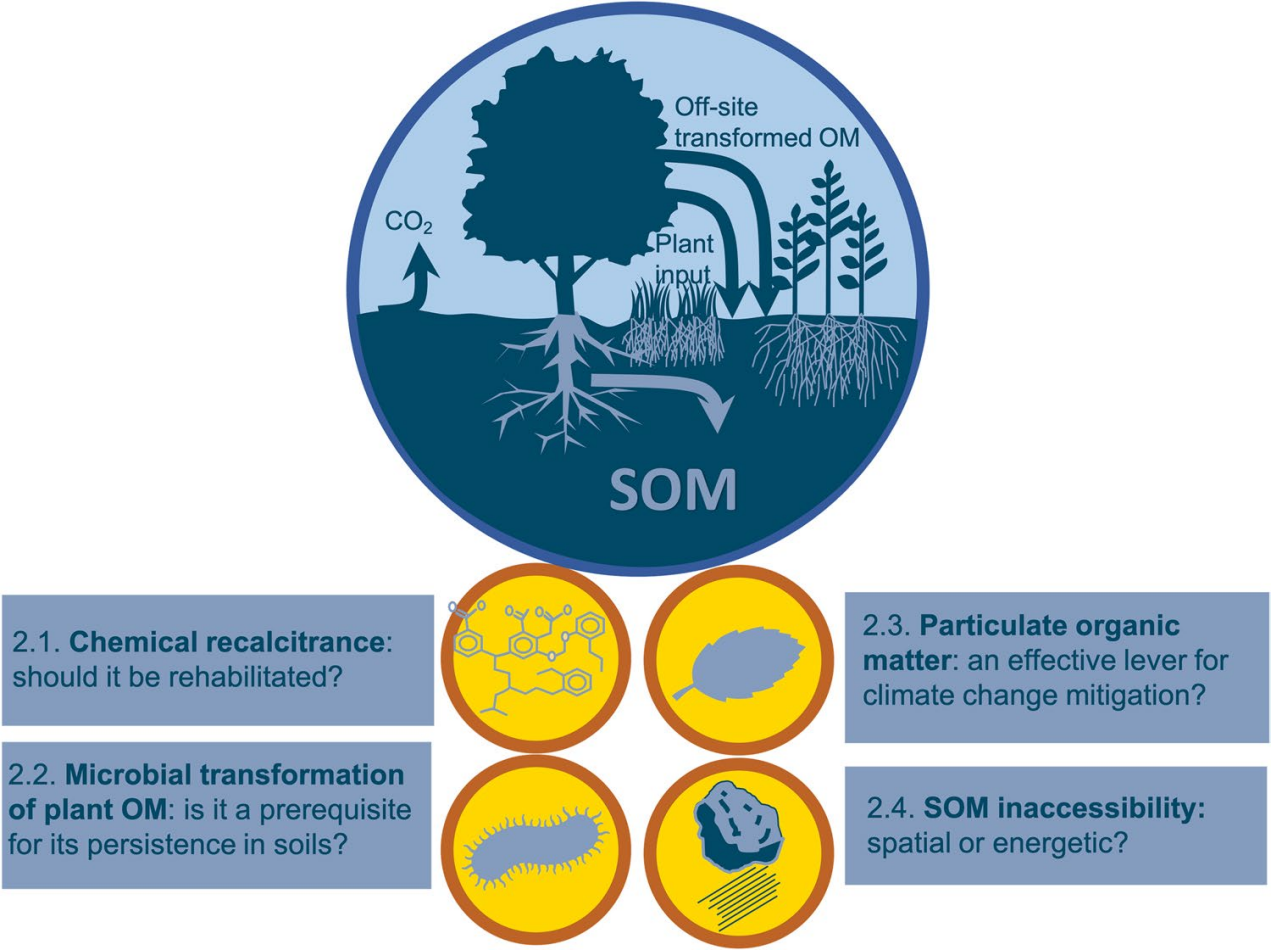
Controversial topics related to the mechanisms that control C storage in soils. (SOM: soil organic matter, OM: organic matter). Recalcitrance has long been considered the major process driving OM persistence in soil, but this has been questioned by a large number of publications in the last decade (section 2.1). A debate has emerged on the nature of persistent C. Often considered as microbe-derived, can it be also plant-derived (Section 2.2). The relevance of particulate organic matter (POM), which contains relatively young C, for accumulating additional C in soil has been recently debated (Section 2.3). Two main theories are discussed in the literature on the factors controlling C dynamics in soil: spatial inaccessibility and a theory based on the bioenergetic constraints of SOM degradation, which we call “energetic inaccessibility” (Section 2.4).

**Fig. 4:**
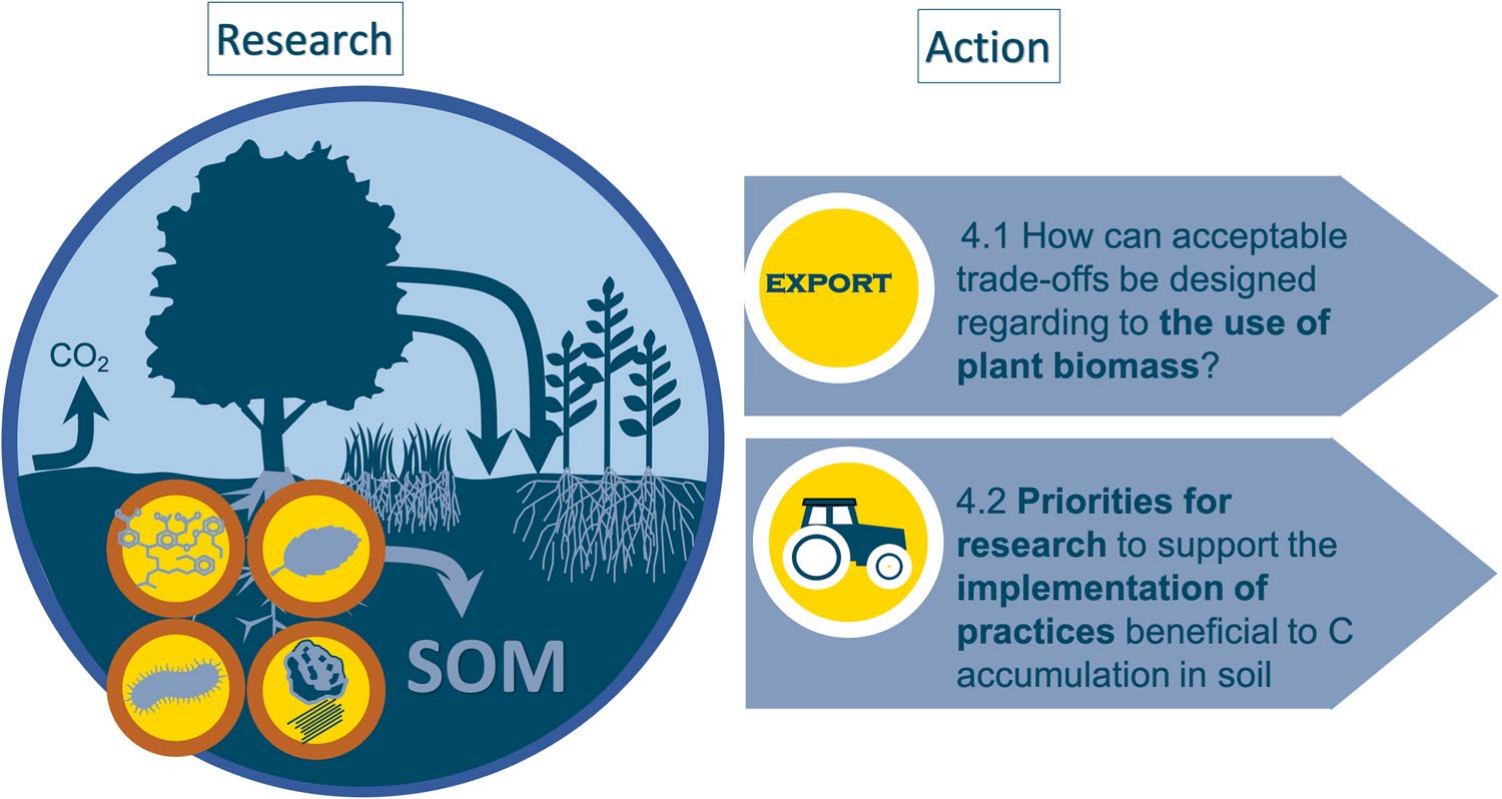
Linking research questions on C storage mechanisms to operational questions.

This review addresses the subject of C storage in agricultural and forest soils by adopting an original approach: it highlights various differences of opinions and proposes some opportunities for reconciliation as well as orientations to support decision-making despite the controversy. Our aim is not to be exhaustive, but to stimulate debate and research in critical areas. In the first part, we describe some current disagreements regarding the mechanisms of C accumulation and loss in soil (Fig. 3). The second part examines the advantages and disadvantages of processing massive datasets and using mechanistic models, with the goal of synthesizing knowledge on C storage mechanisms and generating predictions of C stocks for discussion with stakeholders. The third section examines the main controversies regarding biomass harvest, as well as agricultural and forest management practices that are recommended for increasing soil C stock. It provides advice and orientation for scientific action to better support practitioner and policy-maker decisions (Fig. 4).

## Controversial issues and new challenges in research on mechanisms controlling C storage in soil

Additional C storage in agricultural and forest soil results either from additional C inputs or from increased preservation of soil organic carbon (SOC), which reduces C loss from soil. Both types of processes have a positive effect on climate change by alleviating the amount of C transferred from the soil to the atmosphere as CO_2_ or CH_4_ and should therefore be favored by soil management practices. However, there is a lack of consensus in the scientific community on the mechanisms leading to C storage. In this section, we discuss the currently conflicting views on (1) the notion of chemical recalcitrance, (2) the importance of microbial transformations for C persistence in soil, (3) the contribution of particulate organic matter (POM) to additional C storage, and (4) the preservation of organic matter (OM) in soil due to its spatial or energetic inaccessibility (Fig. 3). We finally conclude with some opportunities for reconciliation among the controversial theories.

### Chemical recalcitrance: should it be rehabilitated?

**Fig. 5:**
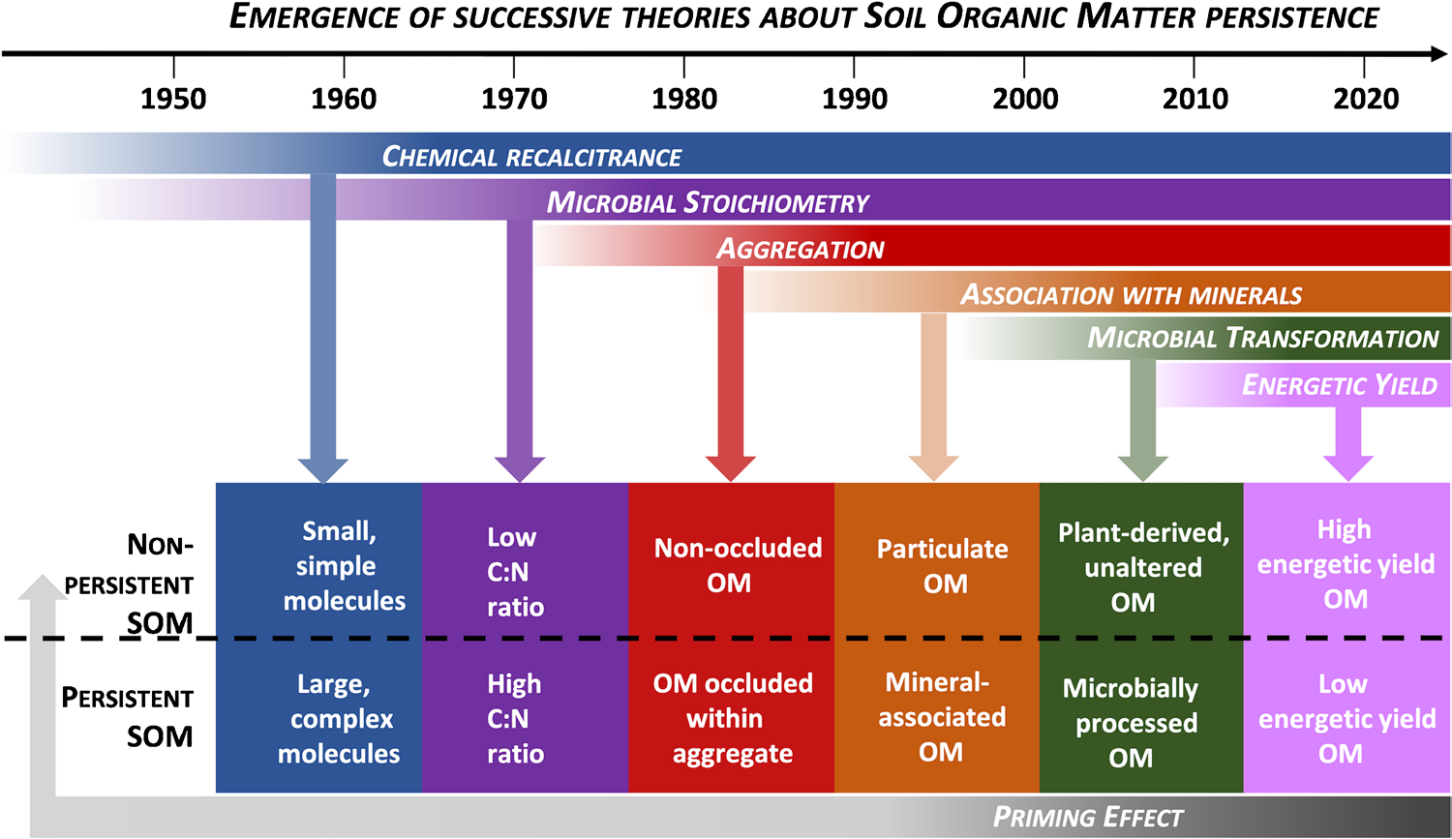
Timeframe of the emergence of the different theories about OM persistence. These theories are not mutually exclusive and can, in most cases, be reconciled. In most ecosystems, several processes act at the same time. In addition, priming appears as a cross-cutting theory, which can explain the shift from persistent to non-persistent SOM. Some theories appear contradictory, such as the persistence of microbial products suggested in the 2000s and the recalcitrance theory suggesting that simple, small molecules are not persistent (see Section 2.2). This does not necessarily invalidate one or both of the theories but highlights the importance of environmental conditions in shaping these processes.

Different definitions of “recalcitrance” can be found in the literature. The most commonly accepted definition in soil science, which we adopt here, is the intrinsic chemical property of a molecule that makes it resistant to decomposition (Kleber et al. 2011; Sollins et al. 1996). Chemical recalcitrance is usually associated with complex and/or polymerized structures (Bertrand et al. 2006; Marschner et al. 2008). These recalcitrant structures may be (i) initially present in plant litter, such as lignin aromatic structures and some aliphatic structures (e.g., Berg 2014), (ii) formed during decomposition (Stevenson 1994) or (iii) formed during thermal degradation (e.g., charcoal formed by wildfires). Recalcitrance is one of the older theories about C persistence in soil (Fig. 5) and has long been considered the main process driving the persistence of OM in soil. However, in recent decades, this concept has been questioned by a large number of publications (e.g., Dungait et al. 2012; Kleber 2010; Kleber and Johnson 2010). We discuss here the approaches that can be used to identify recalcitrant OM, the issues that have made this concept questionable, and whether it can be rehabilitated.

#### A concept partly based on questionable methods

Two methods have historically been used to characterize chemical recalcitrance: litter incubations and measurements of C age in soil organic matter (SOM) fractions (Marschner et al. 2008). Both methodologies have demonstrated a link between chemical structures and C dynamics. However, the C age method estimates recalcitrance on the basis of field experiments examining a timescale of several decades or even centuries, whereas the litter incubation method assesses recalcitrance on the scale of months to years, often in laboratory conditions. The first limitation of the concept of recalcitrance is thus that it is based on methodologies that refer to different time scales, which prevents the development of a single coherent narrative.

A major debate has arisen around the recalcitrance concept in the 2010s with the criticism of the humification theory. According to this theory, products of the degradation of organic substrates are recombined into complex polymers enriched with aromatic functional groups, called humic substances (Schulten and Leinweber 1996). Initially, humic substances were isolated using a soil chemical extraction technique designed to separate organic from mineral compounds (Stevenson 1994). The isolated fraction was found to be enriched in aromatic compounds containing ^14^C that was old in age and was interpreted as recalcitrant. Such results are now widely questioned because, with this extraction protocol, it is possible to generate artifacts in the form of supramolecular assemblies that form under these very specific chemical conditions and are therefore not representative of soil compounds (Myneni 2019; de Nobili et al. 2020; Olk et al. 2019; Sutton and Sposito 2005). Furthermore, despite the use of spectroscopic techniques with very fine-scale resolution, the existence of complex polymeric humic substances in soil has not been proven and some authors have suggested that the thermodynamic investment for decomposers to condense hypothetical “humic substances” would be too high (Kleber and Lehmann 2019; Lehmann and Kleber 2015).

Nevertheless, the theory of SOM condensation still has its defenders who argue that complex structures may naturally form through the condensation of certain organic compounds via the influence of reactive oxygen species generated by brown rot saprotrophic fungi (Goodell et al. 2017; Yu and Kuzyakov 2021) or by mineral phases under specific conditions (Kleber et al. 2021). However, the contribution of these observed condensation processes to the genesis of persistent OM in soil remains to be elucidated.

#### A concept partly based on misunderstood results

At the same time as the concept of humification was called into question, a growing number of studies were quantifying the mean residence time of C in specific molecular structures. In most molecules, the mean residence time of C was found to be similar to that detected in bulk soil (Amelung et al. 2008; Schmidt et al. 2011), contradicting the idea of a specific recalcitrance of some compounds. Even lignin C, which was suspected to persist in soil, was shown to turn over more rapidly than bulk SOC and to exhibit shorter mean residence times (Amelung et al. 2008; Dignac et al. 2005; Gleixner et al. 2002). The only reported exception was pyrogenic C (Schmidt et al. 2011).

This paradox can be explained by several biases encountered in studies of C turnover time in soil molecules. First, an individual biochemical family comprises a very large number of molecules with diverse properties. For example, in the lignin family, the chemical structures of wood lignins are very different from those of the stems of annual plants. Thus, the residence times obtained for C in lignin extracted from maize (rapid turnover, Dignac et al. 2005) cannot be generalized to lignin molecules from other plant species. Then, only some of the members of a given molecular family can be analyzed, i.e., those that can be isolated by wet-chemical methods and that are analytically detectable. It is generally assumed that their C residence time is representative of that of the whole family, which is highly debatable. Another bias is related to the misuse of certain models to assess the mean residence time of C in specific compounds (Derrien and Amelung 2011). Typically, models assessing C mean residence time in microbial compounds must also take into account the initial transit of C in a plant residue, what is not always done. Similarly, there is often a confusion between the age of a molecule and the age of C in that molecule. As an example, let us consider a tree that is several hundred years old. When it dies, the fungi that degrade its wood use the “old” C to create new molecules. The C in these molecules is much older than the fungal molecules themselves and its age is completely unrelated to their recalcitrance. For this reason, one should not claim that a molecule with old C atoms is a recalcitrant molecule. It is necessary to separate the concept of recalcitrance from C age (Kleber et al. 2011): the old C in a molecule may have been recycled from another molecule, and this process may have occurred many times (Derrien et al. 2006).

#### Revisiting recalcitrance in light of other persistence processes

The concept of chemical recalcitrance has also been challenged in the last couple of decades by a considerable number of studies investigating other processes of SOM dynamics (Fig. 5) (Ekschmitt et al. 2005; Schimel and Schaeffer 2012). In particular, a significant research effort has been made since the late 1990s to understand how OM can be preserved through association with mineral phases, which reduces accessibility to decomposers (Kravchenko and Guber 2017; Panettieri et al. 2017; Virto et al. 2010). At the same time, microbial ecology has also provided new insights by showing that OM breakdown depends on the interplay of different microbial communities characterized by their functional diversity (Fanin et al. 2016; Schneider et al. 2012; Bardgett and Van Der Putten 2014). OM is degraded by a succession of microorganisms producing extracellular enzymes that progressively depolymerize and oxidize molecules, releasing small organic compounds and nutrients available for microbial uptake (Amin et al. 2014; Sainte-Marie et al. 2021; Schneider et al. 2012). Although most of the soil enzymes are redundant in terms of the attacked biochemical classes, enzymes greatly differ in terms of structure. As a result, each enzyme breaks some specific bonds with a particular efficiency. The nature of persisting SOM in turn depends on microbial diversity, which controls enzyme functional diversity. Additionally, the activation energy necessary to access and decompose a molecule can be impossible to provide for some microorganisms but provided by others (see Section 2.4). For example, white and brown rot fungi produce enzymes capable of attacking lignins in litter, which most soil microorganisms are unable to do (Floudas et al. 2012; Janusz et al. 2017). Nevertheless, all of the known processes that favor the persistence of SOM (Fig. 5) are related to OM chemistry. For example, the nature of the functional groups in OM governs its interactions with the protective mineral phases, as well as with other organic compounds in the soil. To summarize, the intrinsic properties of organic molecules undoubtedly play a role in C dynamics in soil, but they need to be considered together with the biotic and abiotic parameters of the environment. For this reason, the term recalcitrance should no longer be applied to qualify the global dynamics of a molecule in soil; instead, the term persistence should be used.

#### How can recalcitrance be rehabilitated, and how can it be used in a practical way?

As discussed above, the chemical properties of a molecule are not the predominant driver to explain its dynamics, especially in consideration of different sites with contrasting biotic and abiotic properties. However, for a given set of pedoclimatic conditions, molecule properties do have an influence (e.g., in Versailles, France, different turnover for C in lignin and plant polysaccharides, Derrien et al. 2006; Dignac et al. 2005). The long residence time of the condensed structures of pyrogenic OM in soil (Lehmann et al. 2015) is another good reason to avoid sweeping away recalcitrance.

To rehabilitate the controversial concept of chemical recalcitrance, we propose to define distinct scales of recalcitrance that are applicable in the major pedoclimatic contexts. Practically, a set of targeted pedoclimatic conditions should be identified, encompassing various ranges of nutrient availability, mineral phase properties, decomposer needs and functionalities, and so on. The operational calibration of these recalcitrance scales should be based on standardized degradation tests carried out in the selected pedoclimatic conditions for various molecules/substrates at monthly to pluriannual timescales. Such recalcitrance scales could be disseminated to stakeholders in order to support the selection of additional organic inputs to the soil that favors SOC accumulation under local pedoclimatic conditions (see Section 4.2). They could also contribute to the improvement of Earth System Models by prompting updated versions that go beyond the old concept of recalcitrance.

### Microbial transformation of plant OM: is it a prerequisite for its persistence in soil?

The plant or microbial origin of OM persisting in soil on a multidecadal scale is still a topic of much debate. It was long assumed that persistent SOM mainly originates from plant inputs and forms according to mechanisms that depend on the biochemical quality of these inputs. Some plant biopolymers, such as lignins and long-chain lipids, were thus considered slowly degradable or recalcitrant (see Section 2.1). However, studies conducted over the last decade have suggested that microbial compounds are a major contributor to persistent organic C, and currently, the prevailing opinion is that microbial transformation is a prerequisite for SOM stabilization (e.g., Dynarski et al. 2020; Kallenbach et al. 2015; Liang et al. 2019). We review here how microbial compounds persist in soil despite being theoretically easily assimilable by decomposer. We also present evidence of contexts that lead to the persistence of plant compounds and discuss the preferential use of preserved plant or microbial compounds by decomposers.

#### How microbial compounds are preserved in soil

Since the early 2000s, technological advances in molecular analysis have improved our knowledge on the chemistry of persistent SOM (Kögel-Knabner 2002; Amelung et al. 2008; Gleixner 2013). Using these new techniques, some studies have argued that the molecular composition of persisting OM is closer to that of microbial constituents than to that of plant tissues (Clemmensen et al. 2013; Kallenbach et al. 2016; Liang et al. 2017; Liang et al. 2019; Miltner et al. 2012; Wang et al. 2021). Furthermore, it has been shown that persisting OM is preferentially found in fine soil fractions (von Lützow et al. 2007), which are known to be enriched in compounds derived from microbial OM (e.g., Kopittke et al. 2018).

To understand the mechanisms that could explain the persistence of microbial compounds, we first need to examine their chemical nature. Products of microbial metabolism are exuded and excreted by active microbes or released after cell death (Hofman and Dušek 2003), and range from low-molecular weight and soluble compounds (such as simple sugars, organic acids, and amino acids), to proteins and storage and structural polysaccharides (such as starch and chitin). In theory, these structures are not particularly “recalcitrant,” that is, not complex in their chemical structure (see Section 2.1) (Hopkins and Dungait 2010; Lorenz et al. 2021; Malik et al. 2016). In vitro decomposition experiments under controlled conditions have shown that they may decompose very rapidly (sometimes in less than an hour), probably because they are rich in energy or nutrients, easily accessible to organisms, and rapidly assimilated (Boddy et al. 2007; Hill et al. 2008).

There are several explanations for the apparent paradox of non-complex molecules remaining in soils for a very long time. First, we must remember (Section 2.1) that the age of the C in a microbial molecule does not reflect the age of the molecule, which may be constituted of C that was recycled from other molecules. Nevertheless, it has been argued that the large contribution of microbial compounds to SOM only makes sense if they are somehow protected from continuous microbial recycling (e.g., Derrien et al. 2006). This protection could be due either to chemical or physico-chemical bonding to minerals or to physical occlusion in small aggregates, metal oxides, or short-range-ordered minerals (Kögel-Knabner et al. 2008; Berhe et al. 2012). OM that becomes physically or chemically inaccessible as a result of association with minerals is referred to as mineral-associated OM (MAOM). This is a finer, heavier fraction of OM, which, in addition to the larger and/or lighter particulate organic matter (POM), constitutes the bulk of SOM. We adopt this definition of MAOM in this paper, but must point out that other authors use a more restrictive definition of MAOM that only includes OM chemically bound to mineral surfaces and excludes OM physically protected in aggregate (see Section 2.3). Molecules biosynthesized by microbes have a stronger affinity for protective mineral phases than plant compounds (principally because of their polarity, Kleber et al. 2021), which could be a reason for their large contribution to persisting OM (Cotrufo et al. 2015; Hatton et al. 2012; Kallenbach et al. 2015; Kallenbach et al. 2016; Liang et al. 2017; Miltner et al. 2012; Rillig 2004; Traore et al. 2000). When associated with mineral phases, microbial compounds become less accessible to decomposer enzymes and uptake by decomposer (see Section 2.4). Moreover, accessible mineral surfaces are hot spots of microbial growth and, thus, hot spots of preserved microbial necromass in the form of MAOM (Uroz et al. 2015; Witzgall et al. 2021). Finally, the persistence of microbial compounds could be related to their lower energy content compared to plant compounds, as measured using thermal analysis (see Section 2.4). Indeed, under anaerobic conditions, certain microbial lipids with a low oxidation state might not be used by decomposers due to energetic restriction (Keiluweit et al. 2016).

#### How plant compounds are preserved in soil

The theory that OM persisting in soil is mainly composed of microbial metabolites is contradicted by numerous observations at long-term experimental sites of partially decomposed plant organic residues that persist several decades after their input into the soil (e.g., Amelung et al. 2008; Barré et al. 2018). These studies demonstrate that both microbial and plant-derived compounds contribute to persistent SOM.

**Fig. 6:**
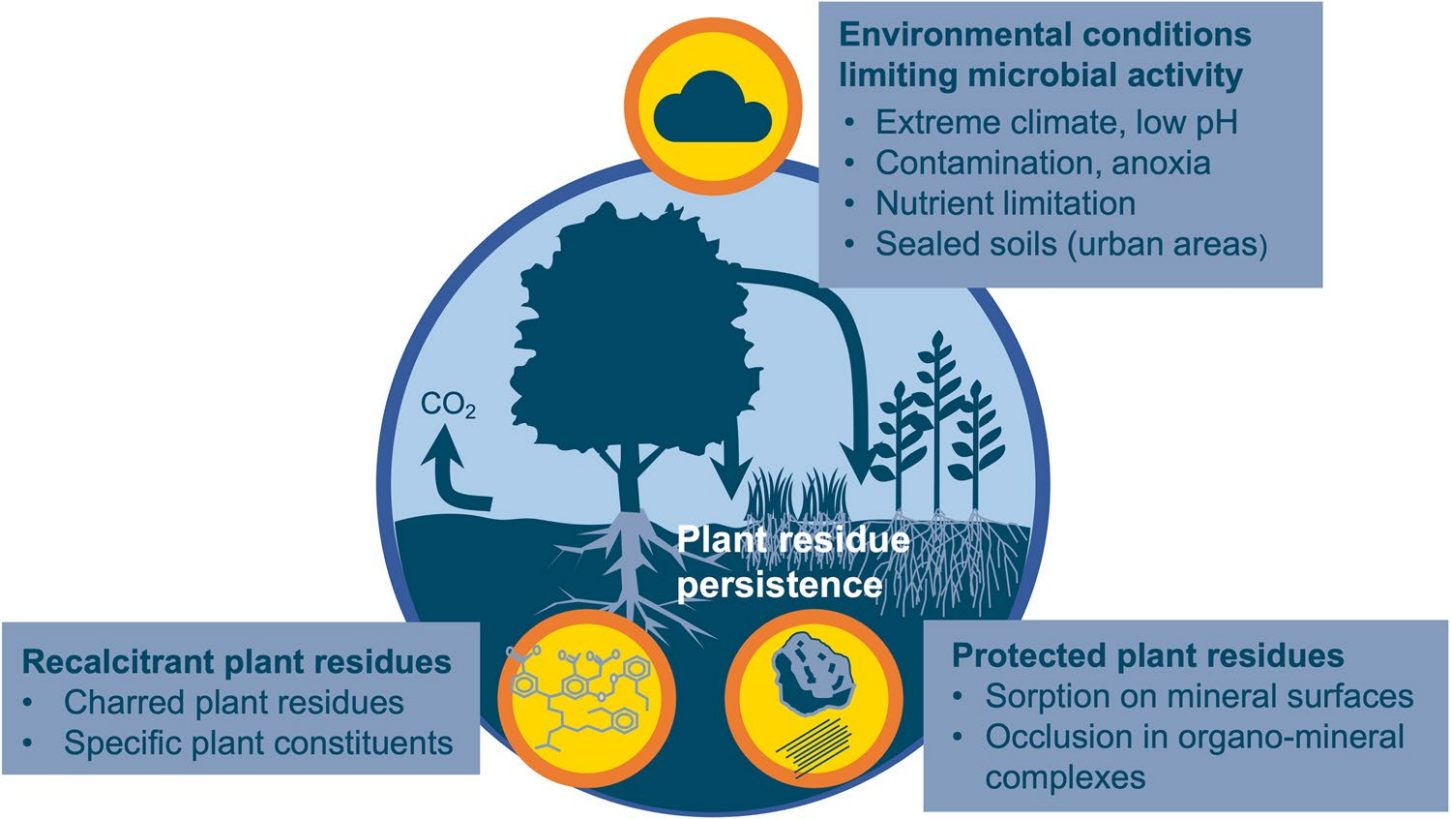
Examples of conditions and processes leading to plant residues persistence.

In some ecosystems, the persistence of OM of plant origin in soil may be due to environmental conditions that limit microbial activity, such as anoxia, lack of water, low temperature, or acidic pH (e.g., Keiluweit et al. 2016; Trumbore 2009) (Fig. 6). This is the case in alpine soils (Budge et al. 2011), high-latitudes soils (Kohl et al. 2018), peatlands (Leifeld and Menichetti 2018), permafrosts (Pengerud et al. 2017; Schuur et al. 2008), and built-up or urban soils (Allory et al. 2022; Cambou et al. 2018; Rees et al. 2019). As an example, in soil submitted to anoxia, the decomposition of certain plant lipids and aromatics could be thermodynamically hampered—as mentioned above for microbial lipids (Keiluweit et al. 2016) (see Fig. 6 and Section 2.4).

The persistence of plant residues may also be due to their association with protective mineral phases. A meta-analysis by Angst et al. (2021) indicated that 50% or more of MAOM (in the broad sense defined above) would be of plant origin, based on the quantification of amino sugars as biomarkers of the microbial-derived OM. The formation of MAOM of plant origin derives from the direct occlusion of particulate plant OM within microaggregates (Lehmann et al. 2008; Virto et al. 2010), which is promoted by detritivore activity (Lavelle and Spain 2001) (Fig. 6). Plant-derived MAOM may also form via the interaction of reactive mineral surfaces with soluble plant-derived compounds (e.g., rhizodeposits, forest floor leachates) (Hagedorn et al. 2015) or with plant polymers transformed by enzyme oxidation or depolymerization that increase their reactivity toward mineral phases (Kleber et al. 2015; Liang et al. 2017; Liang et al. 2019; Wang et al. 2017). This association with protective minerals can occur as soon as plant OM enters the soil. In particular, plant belowground inputs in the form of root tissues or exudates may penetrate aggregates (Freschet et al. 2018; Rasse et al. 2005; Poirier et al. 2018).

The relative contributions of microbial and plant products to SOM are influenced by both land use and soil type (see Section 4.2). The contribution of microbial compounds is reported to be greater in grassland soils as well as in fertile Chernozems or Luvisols (Angst et al. 2021). Instead, plant compounds predominate when soil conditions are less favorable to microbial growth or in coarse-textured soils characterized by organo-metal complexes or short-range-ordered minerals, such as forest soils, Podzols, Ferralsols, Gleysol, or Alisols (Angst et al. 2021; Hall et al. 2020; Kögel-Knabner and Amelung 2021). Furthermore, the overall contribution of MAOM to C storage also varies among soil types (see Section 2.3).

Thus, evidence from the literature of the preservation of both microbial- and plant-derived compounds contradicts the view that microbial transformation is a prerequisite for OM preservation in soil. To improve our understanding of the preferential persistence of microbial or plant-derived OM, we should further investigate patterns of their preferential utilization by decomposers to meet energy or nutrient needs (Bernard et al. 2022) and the likelihood of random encounters between soluble substrates and decomposers (Don et al. 2013; Dungait et al. 2012). There is notably a significant knowledge gap on how variations in micro-environmental conditions affect the accessibility of different OM sources (Bailey et al. 2020) and the energy gained by microbes through their consumption (see Section 2.4).

### Is particulate organic matter an effective lever for climate change mitigation?

The POM fraction refers to organic soil particles that are not associated with minerals. POM consists of coarse plant debris, characterized by a turnover that is generally faster than that of bulk SOM and rather young C (generally a few years old) (Antón et al. 2022). Therefore, POM may not seem to be the most relevant fraction for accumulating additional C in soil (Dynarski et al. 2020). However, one may still question whether POM accumulation could make a meaningful contribution to climate change mitigation. Here, we review the POM properties, its contribution to SOC stock, and its mean residence time, before examining the detailed arguments supporting the use of POM as a means of climate mitigation.

#### Properties of POM

Before discussing properties of POM, an important distinction should be noted. POM has been defined either as a conceptual pool that complements MAOM (see Section 2.2) (Lavallée et al. 2020) or as an operational pool (Poeplau et al. 2018), which is isolated by density or size combined with density, using physical fractionation techniques (typically, density < 1.4–1.8 g cm^-3^ and size > 50 μm) (Hénin and Turc 1949; Leifeld and Kögel-Knabner 2005; Poeplau and Don 2013; Poeplau et al. 2018). The difficulty of measuring conceptual pools and the diversity of fractionation protocols (Lavallée et al. 2020) have led to variation in the properties of POM and MAOM among studies and have impeded generic characterization**.** Consequently, scientists must ensure in their studies that they clearly explain how they define POM and MAOM (see also Section 2.2.1). In the following, POM is considered to be mineral-free and to mainly consist of decomposing plant fragments—with this definition, “occluded POM” is considered as part of MAOM. POM originates from roots and aerial parts of on-site vegetation or from inputs of organic amendments. It is progressively fragmented and incorporated into the soil through the action of living organisms. Pyrogenic C (e.g., charcoals from forest fires) is also found in this fraction (Paetsch et al. 2017). The size of the POM reservoir and its biochemical composition depend on vegetation type, soil biodiversity, and management practices (Section 4.2). Indeed, the magnitude of biomass harvest has a direct impact on plant inputs to soil and on the size of the POM pool (Section 4.1).

#### Contribution of POM to soil C stock

The contribution of POM to SOC is highly variable (Georgiou et al. 2022) and is strongly affected by soil type and depth (Kögel-Knabner and Amelung 2021; Soucémarianadin et al. 2019). It is higher in alpine soils, cryosols, and soils with few reactive minerals (Hagedorn et al. 2019; Kögel-Knabner and Amelung 2021; Leifeld and Fuhrer 2009) than in clay soil from temperate low-elevation regions. POM also depends on soil use and vegetation cover. At the scale of the European LUCAS Soil Network, POM can account for more than a quarter of SOC in the 0–20-cm layer of forests and grasslands, while this fraction is generally smaller in well-drained arable mineral soils (Cotrufo et al. 2019; Lugato et al. 2021; Wander 2004). In forest soils, the relative abundance of POM depends on tree species and is typically lower under broadleaves than under conifers (Cotrufo et al. 2019). In soils that are relatively poor in OM, C tends to be stored more as MAOM than as POM (Cotrufo et al. 2019). C storage in MAOM would be limited to a maximum saturation value (Cotrufo et al. 2019; Georgiou et al. 2022), while POM, instead, does not appear to be limited. The POM fraction, because of its unlimited size, would therefore be an interesting lever for additional C storage and may represent the main opportunity to increase C storage in soil with few reactive mineral phases. However, this additional C storage capacity of POM is still controversial since the duration of this storage, before POM degradation by decomposers, may represent a limit for its contribution to long-term SOC stock build-up.

#### Residence time of C in the POM fraction

The quantities and biochemical composition of POM are dynamic and transient in nature due to the seasonality of plant litter inputs and fluctuation in microbial activity (e.g., Puissant et al. 2017). Because POM is not physically protected from decomposers, it is often considered readily biodegradable (Baldock and Skjemstad 2000; Cotrufo et al. 2019; Paul 2016). However, the biochemical constituents of POM (e.g., cellulose, lignins, pyrogenic C) may exhibit a range of intrinsic biodegradation properties (see Section 2.1 about recalcitrance). In addition, POM use by decomposers depends on its N and P contents (which are often low), on the energy resources it provides to microorganisms, and on the energy required to break bonds within POM biopolymers (Berg 2014; Schmidt et al. 2011; Soucémarianadin et al. 2019) (see section 2.4). As a result, C residence times in POM can range from months to a few decades (Antón et al. 2022; Balesdent 1996; Derrien and Amelung 2011; Paul 2016). The notable exception to this is POM that contains substantial amounts of pyrogenic C, which often has average residence times greater than 100 years (Bird et al. 2015; Chassé et al. 2021; Lehmann et al. 2015).

The residence time of POM is further modulated by soil and climate conditions: it is increased by conditions unfavorable to microbial decomposition (waterlogged soils, nutrient limitations, anoxia, high latitudes and altitudes, arid zones, deep horizons) (Budge et al. 2011; Leifeld and Fuhrer 2009) and under plant species with inhibitory actions on microbial activity (e.g., soil acidification). Peatlands represent an extreme case where the residence time of C in POM can reach thousands of years (Leifeld and Menichetti 2018).

#### Limits and benefits of POM for C storage in soil

The generally short residence time of C in POM is a strong limitation to its utility for additional C storage over the long term. The sensitivity of POM to environmental changes that affect plant inputs or decomposer activity also carries a risk of C storage reversibility by the effects of climate change (Hagedorn et al. 2019; Rocci et al. 2021). This is the reason why some calculation methods of C sequestration potential at regional and national scales (e.g., Alvarez and Berhongaray 2021; Chen et al. 2018) do not consider the accumulation of C in POM but rely only on the finite capacity of the fine soil fraction to sequester SOC in the long term, as first conceptualized by Hassink (1997). However, estimating SOC storage potential only from the storage capacity of the fine fraction, in which C is assumed to persist but to reach saturation, is still a matter of debate. Some research has described MAOM as a composite fraction with some components turning over rapidly on a decadal scale (Schrumpf et al. 2021; Virto et al. 2010), while other authors have argued that soil sequestration potential is primarily determined by inputs rather than by the finite capacity of minerals to accumulate C (Barré et al. 2017).

Nevertheless, POM possesses several characteristics that are advantageous for its accumulation in soil. First, its rapid response to changes in land use and management (Lugato et al. 2021) and its potential pool size—which, unlike MAOM, is not limited—make it a relevant reservoir that can be mobilized in the short term for additional C storage via the implementation (and maintenance over time) of adapted practices (see Section 4.2). Second, increasing C storage in soil in the form of POM would not require additional immobilization of nutrients, in contrast with MAOM, for which stoichiometric requirements can act as limiting factor (see Section 4.2). Finally, the loss of free POM does not mean that its C has been lost from the soil: it may simply move to other compartments of SOM, such as MAOM. For this reason, in all conceptual models of SOM formation—such as humification, selective preservation, microbial decomposition, or the soil continuum model (Basile-Doelsch et al. 2020; Lehmann and Kleber 2015)—POM is involved at the very beginning of persistent OM formation, which it feeds through different mechanisms. Soil faunal activity can lead to the inclusion of POM in soil aggregates (Angst et al. 2019; Le Mer et al. 2020; Six et al. 1999; Vidal et al. 2019). In such circumstances, the age of C in trapped POM (“occluded POM” or “intra-aggregate POM”) can be older than the average age of the SOC (Virto et al. 2010). Some of the C in free POM is also converted to microbial metabolites that have a strong affinity for mineral surfaces and can be preserved over the long term in organomineral assemblages (e.g., Hatton et al. 2012; Six et al. 2006) (see section 2.2). Management practices must promote, as much as possible, this flow of C from unprotected POM to protected MAOM (Kallenbach et al. 2019). More generally, the fact that POM serves as hotspots of microbial activity can drive the formation of organomineral associations. The exopolysaccharides and hyphal network produced by POM-consuming microbes glue together finely sized minerals and constitute a nucleus for aggregate formation and soil C persistence (Witzgall et al. 2021).

### Is soil organic matter persistence driven by spatial or energetic inaccessibility?

Regarding the factors that control C dynamics in soil, two main theories have been recently presented in the literature (Fig. 5): one based on spatial inaccessibility (Chenu and Stotsky 2002; Pinheiro et al. 2015) and the other based on the bioenergetic constraints of SOM degradation (Fontaine et al. 2007; Barré et al. 2016), which we will refer to here as energetic inaccessibility. Whether spatial or energetic, limitations on SOM degradation correspond to the difficulty of substrate use by decomposers. Is it possible to reconcile these two theories? In this section, we define the spatial and energetic inaccessibility of soil C and examine their drivers. We discuss whether spatial inaccessibility may be examined from an energetic perspective and also consider nutrient inaccessibility as a third possible factor controlling soil C dynamics.

#### Spatial inaccessibility

A substrate might be spatially inaccessible as a result of two distinct processes: (i) its inaccessibility to decomposer enzymes and (ii) its impossible uptake by decomposers while it occurs as oligomer or monomer. Spatial accessibility is an issue at both the nano- and microscales, e.g., through the adsorption of OM onto mineral phases or its entrapment in environments with reduced pores, such as soil microaggregates or mineral-organic coprecipitates (Erktan et al. 2020; Hemingway et al. 2019; Kleber et al. 2015; Kravchenko and Guber 2017; Rowley et al. 2018). It can also be an issue at the macroscopic scale, for example, in the case of a mulch applied to the soil surface that is not in contact with soil organisms (Bleuze et al. 2020). Spatial inaccessibility depends on soil physico-chemical properties that determine the physical protection of OM such as the nature of the mineral phases, pH, the composition, and ionic strength of the soil solution (Newcomb et al. 2017; Sposito 2008), and the soil water content, which controls the diffusion of OM/nutrients and the mobility/motility of organisms in the soil porosity (Kleber et al. 2021). It also depends on the actions of mesofauna and earthworms, which alter pore geometry and connectivity (Erktan et al. 2020).

#### Energetic inaccessibility

Energetic inaccessibility corresponds to an unfavorable balance between the energy invested by decomposers to obtain access to a substrate and the energy that is gained from its mineralization (Fontaine et al. 2007; Kleerebezem and Van Loosdrecht 2010; Rovira et al. 2008; Williams and Plante 2018). This concept is therefore dependent on the properties of a substrate (diversity and number of monomers and strength of bonds between them) that makes it either thermodynamically expensive or appealing for decomposers (Lashermes et al. 2014; Lashermes et al. 2016; Moorhead et al. 2013).

This cost/benefit ratio can be estimated by thermal analysis (Dufour et al. 2021; Williams and Plante 2018). The cost corresponds to the activation energy required to overcome the energy barrier for mineralization; this is evaluated by the stability of a compound subjected to a temperature increase. It can also be assessed using incubations that aim to quantify the substrate decay rate at different temperatures, in order to calculate the activation energy as a proxy of the energy cost (e.g., Leifeld and von Lützow 2014; Hemingway et al. 2019). The benefit corresponds to the energy released during mineralization of the compound (e.g., by respiration) and is measured by calorimetry. Several studies have reported that the energetic characteristics of persistent SOC are different from those of fresh SOC. Persistent SOC tends to be more thermally stable and its combustion generates less energy (e.g., Barré et al. 2016; Hemingway et al. 2019; Henneron et al. 2022; Plante et al. 2011; Plante et al. 2013). These observations suggest that SOC persists when the cost of its degradation is too high relative to the benefit to microorganisms. Some experimental (Barré et al. 2016; Cécillon et al. 2021) and theoretical (LaRowe and Van Cappellen 2011; Manzoni et al. 2012) studies have indicated that, under oxic conditions, reduced compounds provide more energy to microorganisms than oxidized compounds, and better promote microbial growth. These studies have also suggested that plant-derived compounds provide more energy than microbial compounds in well-aerated soils. In contrast, in anoxic environments, reduced compounds persist because their decomposition would require too much activation energy and their fermentation would yield too little energetic benefit (Boye et al. 2017; Keiluweit et al. 2016).

With respect to the interpretation of thermal data, though, it should be noted that thermal methods are integrative and take into account the total OM of the analyzed sample, whereas under real conditions, decomposers have access to only a small fraction of this OM (Leifeld and von Lützow 2014). Alternatively, energy investment can also be considered as the cost of exoenzyme production (Bosatta and Ågren 1999). In this context, analyses must consider the temporal dimension since microorganisms produce enzymes using previously acquired resources (Amin et al. 2014; Fontaine et al. 2007; Klotzbücher et al. 2011).

#### The energetic dimension of spatial inaccessibility

Spatial inaccessibility may be altered by changes in local environmental conditions. Such modifications can be the result of energetic investment by decomposers in the production of weathering agents (e.g., protons, siderophores), that desorb substrates associated with minerals and increase their accessibility (Henneron et al. 2022). The extent of this investment varies with the type of bond to be broken: more investment is required to break covalent bonds between OM and mineral surfaces than to break electrostatic bonds between oppositely charged organic and mineral ions (Kleber et al. 2015). With an increased understanding of microbe-driven desorption efforts, it may be possible to better integrate the concepts of spatial and energetic accessibility.

#### Nutrient issues, a third mechanism of inaccessibility limiting soil OM decomposition

Beyond the acquisition of C, the benefit of an organic substrate for microorganisms must also be evaluated in terms of acquisition of nutritive elements (e.g., N, P, Ca, Mn). Indeed, nutrients are essential for metabolite production and decomposer growth (Hemkemeyer et al. 2021; Monod 1949; Saadat et al. 2020). OM utilization is thus not just a matter of whether an energy balance is favorable or not for microorganisms, but also, whether the stoichiometric needs of microorganisms are satisfied (Kleerebezem and Van Loosdrecht 2010; Margida et al. 2020; Moorhead et al. 2012; Torn et al. 2005; Zechmeister-Boltenstern et al. 2015). Stabilized SOM has near-constant C:N:P ratios across ecosystems (Bertrand et al. 2019; Kirkby et al. 2013), which could imply that additional C storage would be necessarily associated with nutrient immobilization—a hidden cost of C persistence in SOM (Richardson et al. 2014) (see section 4.2).

Microbes are not completely powerless in the face of soil nutrient depletion; many are able to modulate their metabolism to better forage for nutrients (Bertrand et al. 2019; Recous et al. 2019). For example, microorganisms may produce enzymes to mineralize nutrient-containing SOM, such as nitrogenous OM. This selective mining leads to an acceleration (or priming) of SOM mineralization kinetics (Craine et al. 2007; Fontaine et al. 2011; Shahzad et al. 2015; Hicks et al. 2021). Microorganisms can also retrieve nutrients such as phosphorus, magnesium, calcium, or iron from mineral phases by producing weathering agents such as siderophores or organic acids (Bailey et al. 2020; Keiluweit et al. 2015; Uroz et al. 2009; Uroz et al. 2020). If these mechanisms are not efficient enough to meet the stoichiometric requirements of microbes, though, microbial activity decreases, limiting the decomposition of OM.

**Fig. 7:**
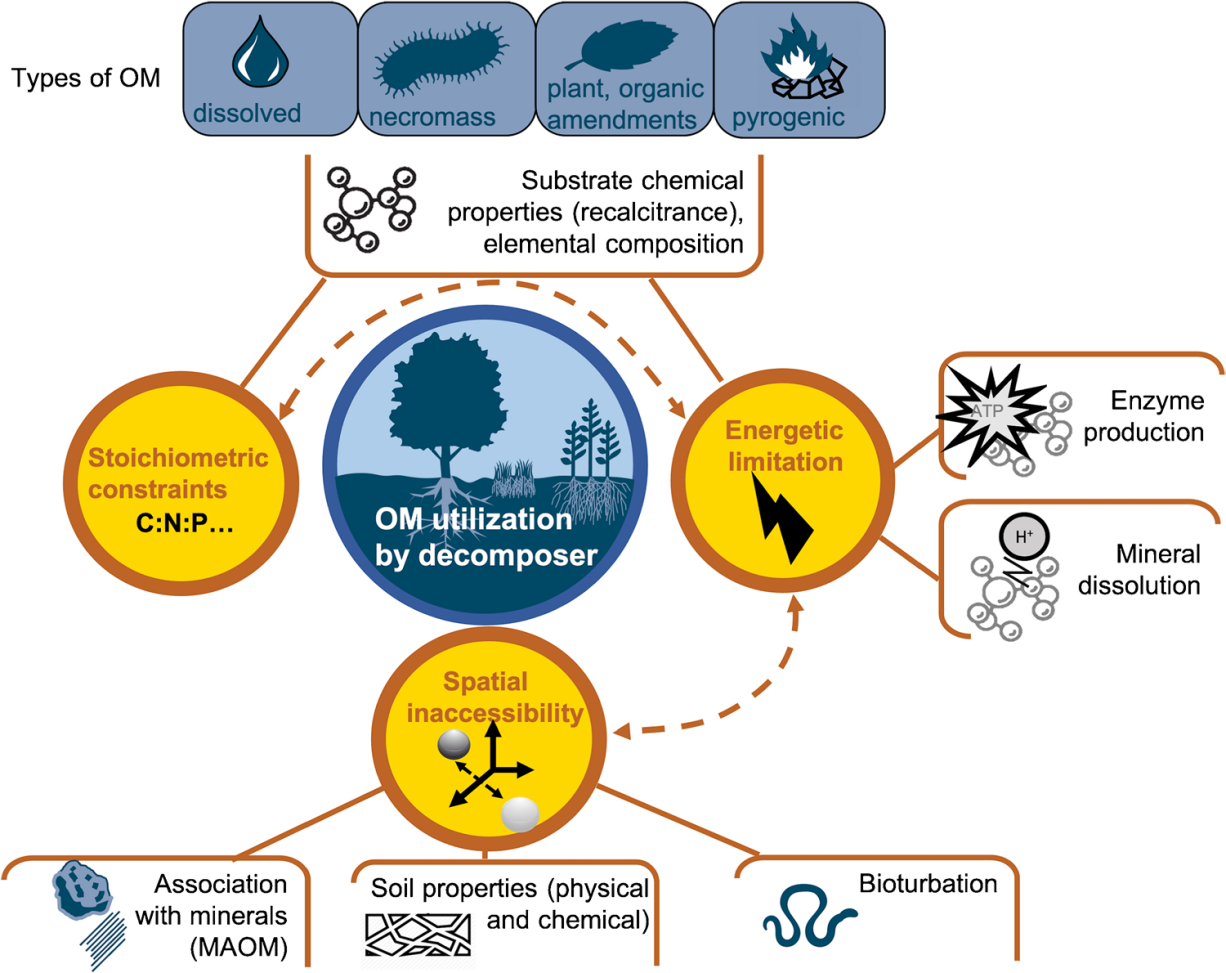
The persistence of OM (whatever its nature: plant-derived, decomposer-derived, pyrogenic or dissolved) results from the conjunction of different mechanisms controlling OM utilization by decomposer. These mechanisms are linked to stoichiometric, energetic, or spatial constraints. The chemical properties of OM drive stoichiometric and energetic limitations. Enzyme production and mineral dissolution drive energetic limitation. Organo-mineral associations, soil properties, and bioturbation by soil fauna drive spatial limitation. Dotted arrows indicate links between constraints.

In conclusion, OM persistence in soil is never driven by purely spatial or purely energetic constrains. A holistic view of these processes must be adopted, with particular attention to the impacts of environmental parameters and practices on inaccessibility processes (Fig. 7).

### Reconciling competing theories of C persistence

In this first part, we have described how the apparent conflict between theories of soil C storage mechanisms is not actually rooted in any definitive antagonism (Figs. 5 and 7). We have shown that recalcitrance is not an all-or-nothing concept, but must be seen as a regulator of OM persistence. Recalcitrance could be defined for each major pedoclimatic context, using a quantitative index in standardized conditions. The debate on the relative importance of microbial and plant origins of persistent OM can also be resolved by considering pedoclimate properties, which control (i) the intensity of microbial activity and transformation of plant OM into microbial OM, and (ii) the presence of reactive mineral phases crucial for the preservation of thermodynamically labile microbial compounds. We have explained how POM could be an asset for C accumulation. Although its rapid turnover and smaller size (relative to MAOM) are detrimental for additional C storage, this effect may be offset by its rapid response to management practices, its potential non-limited size, its stoichiometry, and its ability to feed and promote C preservation in MAOM. Finally, we have clarified that, when environmental properties are considered, theories of SOM inaccessibility—spatial, energetic, or related to nutrient availability—are complementary rather than conflicting, and need to be considered together to explain C persistence in soil.

## Integrating mechanisms into databases and models

Databases and models have important roles to play in the efficient synthesis and dissemination of knowledge on mechanisms involved in SOM storage as well as in the translation of scientific knowledge into recommendations for management practices beneficial to C storage. Nevertheless, caution must be exercised in the use of databases, and the scientific community is still divided on the relevance of increasing the number of processes described in models. We review here the benefits and pitfalls of (1) large databases and (2) process-based models, and conclude with a discussion of the good practices for their use in soil science.

### Big data: opportunity or danger for research in soil science?

Data are produced or collected on a massive scale by researchers, practitioners (Billings et al. 2021), and citizens involved in participatory research. Big data, i.e., the analysis of massively acquired data, offers a complementary alternative to experimental research on mechanisms, which are necessarily limited by cost and/or complexity of advanced analytical techniques. We discuss here the opportunities opened by the increasing availability of soil-related data and underline critical points of caution before proposing a roadmap for big data deployment in soil science.

#### Opportunities of big data

Massive datasets represent a real opportunity to synthesize and make available to the community a wide range of knowledge obtained on large spatial scales (e.g., research observation networks, national or continental scale), as proposed, for example, by international databases on soil respiration or radiocarbon (Jian et al. 2021; Lawrence et al. 2020). The exploration of such massive datasets then serves as a means of generating new insights from multiple data aggregation. For instance, through archiving recently acquired data and digitizing old data, it may be possible to monitor the evolution of soil C stocks and then gain insight into the mechanisms involved.

Another strength of big data is that it can massively combine data on the mechanisms controlling C storage in soil with georeferenced environmental and even socioeconomic data (e.g., pedological, biological, geological, geomorphological, ecological, hydrological, climatic, agronomic, forestry, economic and historical data), thus promoting interdisciplinarity. It can increase our knowledge of mechanisms of soil C storage (Cécillon et al. 2015; Vestergaard et al. 2017) by (1) providing evidence for the genericity of certain processes, or, alternatively identifying pedoclimatic contexts that are characterized by distinct processes, and, by (2) ranking the importance or relevance of soil C storage mechanisms among different types of pedoclimate or plant cover. Statistical methods such as path analyses can be used to infer dependence and causality between environmental variables and soil storage mechanisms (Cotrufo et al. 2019; Lange et al. 2015). In addition, machine learning models can be constructed to directly predict the intensity of soil C storage mechanisms from environmental metadata readily available from online databases (Cotrufo et al. 2019; Sanderman et al. 2017). Machine learning models can also be developed using massive data obtained by applying simple and fast analytical techniques, such as near infra-red spectroscopy, to a large number of samples to predict OM characteristics (e.g., Dangal et al. 2019; Viscarra Rossel et al. 2019). Such characteristics are classically measured by more precise but complex or expensive analytical techniques, which are not accessible to all scientists. The extent of the data collected compensates for the lack of sensitivity of these massively deployed simple methods and enables detection of significant trends.

#### Points of caution associated with big data

With the emergence of big data, soil science and biogeochemistry are facing a profusion of data, which are sometimes inconsistent and may have few quality controls. Moreover, the skills to properly process and interpret such data are scarce. The danger is that the noise associated with inconsistent or non-validated data may prevent the detection of real trends. The deployment and use of big data must therefore be guided by a number of warnings from the scientific community. The pitfalls to be avoided when exploiting massive databases are (i) producing only obvious trends that have been understood for a long time (e.g., soil OM stock is related to clay content); (ii) seeking to detect only global trends, whereas situations outside the global trend may be of particular interest (e.g., risk of overlooking outliers such as a sandy soil with a lot of C, which could provide new information); (iii) aggregating and comparing data that are not comparable because they were obtained using different methods (e.g., soil organic carbon stocks obtained by wet chemistry, dry combustion, or loss on ignition are not equivalent—Tivet et al. 2012); and (iv) inferring irrelevant mechanisms or causality from large datasets (e.g., through misused machine learning without critical evaluation—Wadoux et al. 2020).

With the use of big data, there is also an increase in the distance between the people who use the data and those who carry out the soil sampling, data acquisition, and archiving in databases. Within the field of soil biogeochemistry, we must be careful not to deploy big data at the expense of soil science and metrology, which are and should remain essential pillars of the discipline. Big data also represents a risk for data ownership and access. To avoid uncontrolled exploitation of data, partnerships may be necessary, in particular with private companies.

#### Roadmap for the deployment of big data in soil science

Our community can contribute to several important projects on the implementation and rigorous use of big data. The first relates to the harmonization of data on C storage mechanisms (nature, unit, etc.). For this purpose, the use of international ISO metrological standards is essential when collecting data. Our community can also contribute to the development of new standards (Bispo et al. 2017) (e.g., European INSPIRE Directive; constitution of metadata catalogs; application of interoperability rules; FAIR data principles (Findable, Accessible, Interoperable, Reusable)). Finally, it is necessary to make the database frameworks compatible with future scientific advances to make sure that it will be possible to integrate novel data generated by innovative methodologies (e.g., "-omics") and associate them with new standards.

### Is it necessary to integrate novel mechanistic knowledge in models of SOM dynamics?

**Fig. 8:**
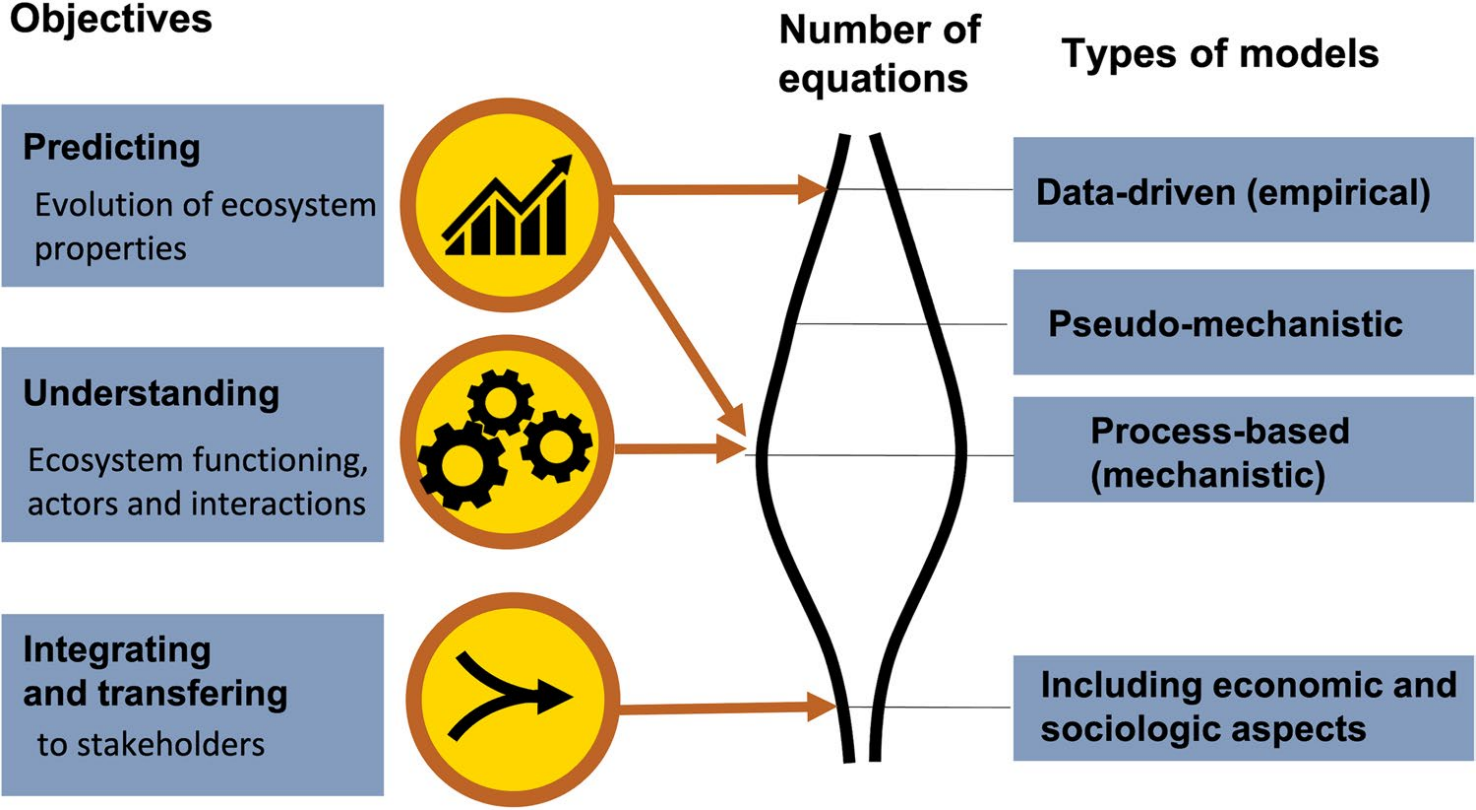
A model’s purpose must be the foremost consideration in its design and the number of equations it includes. There is a continuum of models from data-driven models which include few equations on the one hand to process-based models, which rely on numerous mechanistic equations on the other, and pseudo-mechanistic models that are intermediate between the two end-members.

A model is a simplified representation of reality. It is, however, often very useful for three main purposes in the field of SOC storage: (i) improving scientific knowledge on soil functioning, (ii) providing predictions of C storage in soil, and (iii) supporting policy-makers and practitioners for practices and management choices. A model can be built either on the basis of observations that are reproduced using a reduced number of equations and parameters (data-driven models, also called empirical, statistical or phenomenological models) or by gathering knowledge or hypotheses on soil functioning and translating each process into an equation, which leads to a much larger number of equations and parameters in the model (process-based models, sometimes called mechanistic models). There is also a vast range of “pseudo-mechanistic” models that are intermediate between data-driven and process-based models (see examples below) (Fig. 8). Technical advances in recent years have led to a considerable body of new knowledge on the factors controlling SOM dynamics, particularly regarding the functional diversity of decomposers and the importance of the spatial organization of the soil matrix (Blankinship et al. 2018; Kleber et al. 2021; Lehmann et al. 2020; Miyauchi et al. 2020; Zhang et al. 2016). Will the integration of this new mechanistic knowledge into models improve the accuracy of their projections? In an attempt to answer this question, we first survey the benefits and limitations of process-based models and then discuss how the expectations of the model’s end user should be considered in determining how precisely processes should be described in models.

#### Benefits and limits of process-based models

A detailed description of processes in models can be very useful when the interactions among several processes and/or actors lead to complex dynamics that cannot be analyzed using the usual human deductive reasoning (Waring et al. 2020). Process-based models allow knowledge to be aggregated and simulations to be generated for scenarios, including changing environmental conditions. However, increasing the number of processes described in a model also increases the number of parameters as well as the uncertainty associated with predictions (Shi et al. 2018). Moreover, the acquisition of some of the data needed for parameter calibration and model validation might be experimentally difficult (Allison 2012; Blankinship et al. 2018; Lehmann et al. 2020). For all these reasons, it is very important to respect the principle of parsimony when choosing the mechanisms to be included in a model (Shi et al. 2018).

For models implemented at regional, national, or global spatial scales, the description of numerous mechanisms may require computational resources that are expensive or simply unavailable. In such situations, one solution may be to apply mechanistic models that analyze a limited number of carefully selected soils representative of typical pedoclimatic conditions in order to identify the emerging factors controlling OM dynamics. These determining factors are then introduced into simpler models deployed on a large scale for the same type of sites (Lehmann et al. 2020). Another way to circumvent the problems of process-based models is to create a “model of the model,” a so-called metamodel (Garcet et al. 2006). The metamodel is built by running the initial model in a wide range of conditions and by applying statistical approaches to the simulated outputs to determine new equations that require fewer parameters and a shorter computation time. Such metamodels are, however, more rigid than the original process-based model and may not give relevant predictions in complex, fast-changing environments.

It must also be kept in mind that models reflect the knowledge available at a given time. Models do not report an absolute truth and are likely to evolve with the progress of knowledge but also with the changing expectations of societies. Furthermore, the notion of a “mechanism” is itself complex and dependent on the scale at which the researcher is investigating. For example, a soil scientist identifies mechanisms of ecosystem functioning through observations that are in a large part only the macroscopic manifestation of processes taking place at much finer scales (e.g., molecular interactions, quantum physics) (Allison 2012). These limitations suggest that, despite the considerable amount of progress that has been made, current knowledge of the mechanisms contributing to ecosystem functioning remains incomplete.

#### Toward models adapted to users' expectations

The degree of mechanism that should be incorporated into a model depends on the modeler’s priorities among the three objectives previously defined (i.e., better understanding, scientific predictions, transfer to stakeholders) (Fig. 8):

1. To understand the functioning of a system, process-based (mechanistic) models may represent the best option. Playing with such models (e.g., “toy models” that have been designed to represent a theory or a mechanism in a simplified way) facilitates the identification of the roles of drivers and their interactions. Process-based models have led to significant advances in the understanding of biogeochemical cycles and supported the design of a new generation of experimental work (Barot et al. 2014; Daufresne and Loreau 2001; Moorhead et al. 2012; Perveen et al. 2014; Sainte-Marie et al. 2021; Sulman et al. 2017).
2. To predict alteration in ecosystem processes such as primary production and C storage and to share the results with policy-makers, the use of complex process-based models must be performed carefully. For short timescales (a few years to decades), data-driven models or pseudo-mechanistic models—such as RothC (Coleman and Jenkinson 1996), or AMG (e.g., Clivot et al. 2019; Levavasseur et al. 2020)—are probably the most effective tools. By incorporating only a few parameters and relying on a very large number of past observations, such models guarantee solid predictions in the short term in an environment that does not change much. In unstable conditions, however, the predictions of data-driven models could become questionable (e.g., Waring et al. 2020). When the environment evolves outside the framework for which the parameters were determined, these simple models may no longer be applicable (e.g., Georgiou et al. 2017). By explicitly representing the important processes in a system, mechanistic and pseudo-mechanistic models are more suitable for describing transient phenomena and the transition towards new equilibria (Finke et al. 2019; Keyvanshokouhi et al. 2019), and increase confidence in model predictions (Bradford et al. 2016). This is the strategy implemented in Earth System Models utilized for projections by the Intergovernmental Panel on Climate Change (IPCC) (e.g., Guenet et al. 2018). Moreover, to provide predictions to public policy decision-makers, it may be relevant to use several models based on different assumptions (Wieder et al. 2018). The range of simulations produced by the so-called ensemble modeling method provides uncertainty associated with the estimates (Farina et al. 2021; Shi et al. 2018; Sulman et al. 2018).
3. To integrate and transfer knowledge to stakeholders (students, citizens, politicians, practitioners and policymakers), models describing ecosystem functioning must also include economic and sociological components (e.g., multi-agent models—Bousquet and Le Page 2004). In this way, they can highlight the advantages, disadvantages, and trade-offs of various practices and management scenarios for human society (e.g., Pellerin et al. 2019). These decision-support tools are necessarily very integrative, but this integration comes at the cost of a simplification of the ecosystem mechanisms description. These models must be co-constructed with stakeholders to meet the needs of decision-makers, and with an interdisciplinary scientific community to better objectify the choices to be made for a simplified representation of ecosystems. The scientific community must also estimate the uncertainties associated with the predictions when possible. Finally, particular attention must be paid to the ergonomics of the modeling tool, to ensure that it has an intuitive interface that is easy to use by non-specialists (e.g., serious game, Jouan et al. 2020).

### Databases and models: summary of benefits and pitfalls

We have shown here that big data and models offer a huge potential to improve our understanding of soil functioning, predict C dynamics, communicate and transfer scientific knowledge to stakeholders. Both tools must be used with caution, bearing in mind the benefits and limitations. Scientists must stay alert when developing and using these resources to avoid the pitfalls of database exploration and to optimize the robustness of model prediction. Because big data in soil science is still in its infancy, proper practices can contribute to its successful expansion and implementation. An increase in the number of processes described in a model framework must be justified by the model purpose, as a large number of parameters may be beneficial to knowledge production but detrimental for model use by non-experts. Our community should also favor collaborative exchanges between modelers and researchers who contribute to the production of experimental knowledge on processes in order to support model progress in a direction that is useful to stakeholders and to all scientists, whether experimentalists or modelers.

## Debates on the effects of harvest and management practices: how can research support practitioner and policy-maker decisions?

While research on C storage in soil focuses on how biomass-derived SOM can contribute to climate change mitigation, there is at the same time an increasing demand for biomass harvest, which decreases C input to soil and modifies C stock. Indeed, plant biomass is also central to the global challenges of food security and fossil fuel dependency that need to be addressed simultaneously. In addition, although current knowledge on beneficial management practices has been summarized in several recent reviews (e.g., Amelung et al. 2020; Chenu et al. 2019), the rational mechanisms behind the observed benefits are still poorly understood or intensively debated, and the possible unsuspected interplay between processes may impair the expected effects.

In this section, we provide recommendations for research actions to better support stakeholders’ decisions regarding harvest and management practices, which are still highly debated for their actual contribution to climate change mitigation. First, we address the issue of biomass harvest by analyzing the trade-offs between directing plant residues to the soil and using them for food, energy, or materials. Then, we analyze the areas of contention related to agricultural and forestry management practices recommended for soil C accumulation.

### How can acceptable trade-offs be designed regarding the use of plant biomass?

**Fig. 9:**
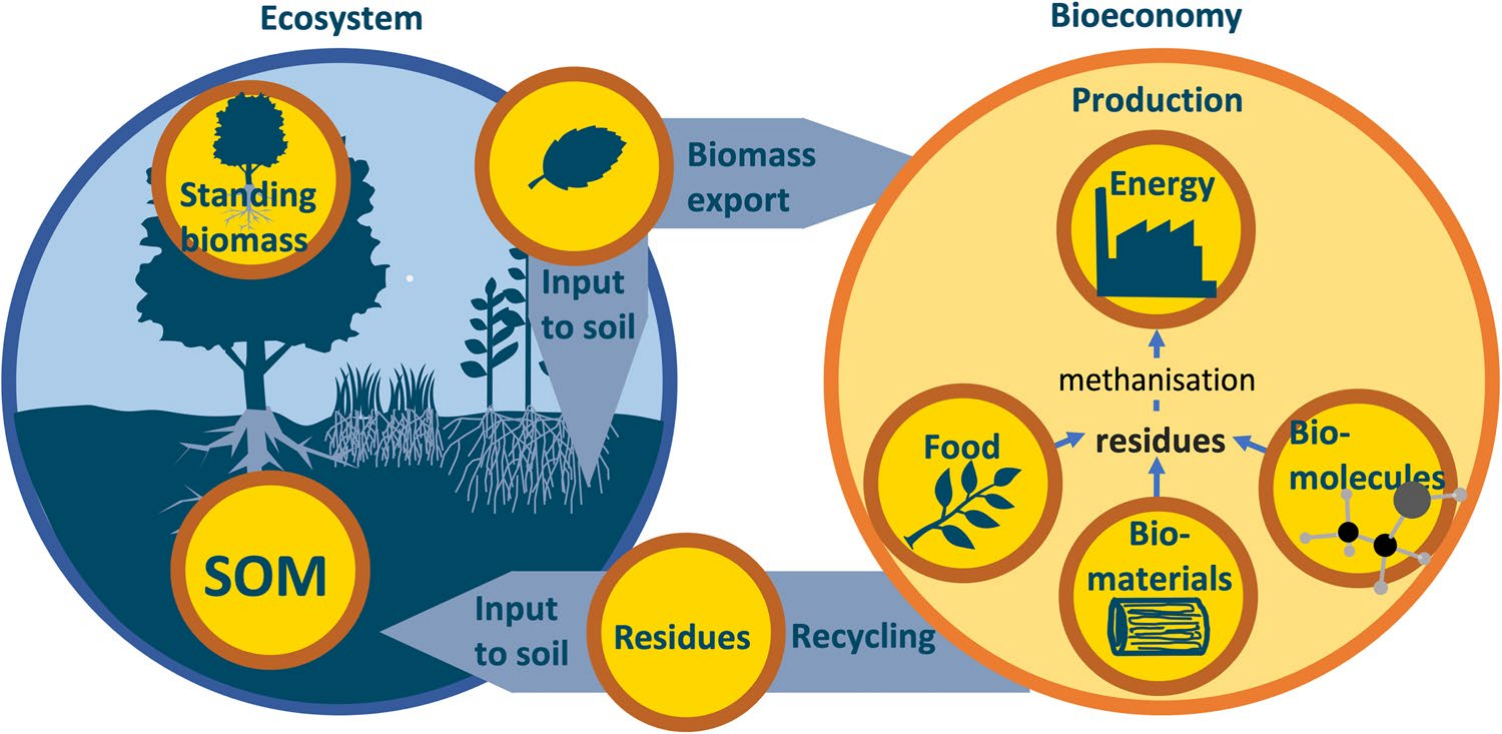
Biomass fluxes among ecosystems and the bioeconomy: exports to food, bioenergy, biomaterial, and biomolecule sectors, return to the soil or standing.

In managed agrosystems and forests, most of the aboveground plant biomass is harvested by humans to produce (i) food, (ii) biosourced energy or (iii) biomaterials and biomolecules (Fig. 9). The above-ground biomass that is not exported contributes to the transient storage of C in living standing biomass and feeds SOM (Fig. 9). Throughout history, biomass harvest has progressively spread over larger surfaces and its intensity has increased. This continuous and increasing harvest of biomass has led to a decrease in soil C stocks compared to a hypothetical world without agriculture (Sanderman et al. 2017).

The current demand for biomass is reaching a critical point. Food demand is projected to increase by 50% between 2012 and 2050 (The Future of Food and Agriculture—Trends and Challenges 2017), as a consequence of population growth. The biomass use for the production of bio-based energy, biomaterials or biomolecules is also increasing with the aim of substituting fossil C (Favero et al. 2020). To meet the needs of the bioeconomy, either the level of biomass harvesting must be increased or the amount of land devoted to agriculture or forestry must be expanded.

When designing trade-offs between exporting biomass or keeping it in the ecosystem, it is important to first take into account the expected duration of C storage. The storage duration in biomaterials is expected to be less than or equal to the one in soil. Indeed, the mean age of soil C is 100 years at 20 cm depth in the tropics (Balesdent et al. 2018), compared to a lifespan of 50 years for wood timber and 4 years for paper (Valade et al. 2018). In the soil C storage, duration can be increased by transforming biomass (e.g., into compost, biochar, methanization residues) before applying it to soil. A second important consideration is that the harvested biomass must have indeed substituted fossil C (Sathre and O’Connor 2010; Amelung et al. 2020), which could be confirmed by life cycle analysis that integrates the C emissions of intensified management, biomass conversion yields into energy or finished products, and off-farm transformation, among other factors (e.g., Valade et al. 2018). From this perspective, the C balance would obviously be more oriented towards C-saving for a territory capable of transforming locally produced biomass into locally consumed energy than for an area importing biomass and exporting the energy produced. Finally, soil fertility and production capacity should be carefully addressed, since biomass export affects not only C stock but also nutrient stocks (Achat et al. 2015; Durante et al. 2019; Legout et al. 2020). Under low fertility conditions, practices that favor the return of harvest residues to the soil should be encouraged. These practices will also participate to the preservation of other ecosystem functions (e.g., water quality and quantity, biodiversity) (Baveye et al. 2020; Hoffland et al. 2020).

### Priorities for research on SOM mechanisms to support the implementation of practices beneficial to C accumulation in soil

Currently certain agricultural and forestry practices are recommended for promoting OM accumulation in soil (Chenu et al. 2019; Dynarski et al. 2020; Lugato et al. 2021; Schlesinger 2022). However, their benefits depend on soil characteristics (Amelung et al. 2020), and their net effect on C stock in the long-time has not yet been analyzed. It is possible that these practices may ultimately have some negative repercussions on climate mitigation or soil quality. Unfortunately, we cannot afford to wait until long-term studies have been performed: immediate action is needed to maximize the potential benefits of soils for climate change as soon as possible (Chabbi et al. 2017).

From a mechanistic knowledge basis, Table 1 summarizes the potential positive and negative effects induced by recommended management practices, categorized according to their targeted action: (1) increase in C input; (2) increase in N input; (3) enhancement of soil life, and (4) increase in spatial inaccessibility.

**Table 1:** Four categories of practices (see also the reviews by Dignac et al. 2017; Chenu et al. 2019; Amelung et al. 2020) with expected positive effects on C storage in soil are distinguished according to the mechanisms targeted: C input (practices that increase the input of C to soil); N input (practices that increase the input of N to soil); biology (practices that aim to maintain microbiological and biological life in soil); inaccessibility (practices that impede decomposers access to organic matter). Expected positive effects and potential negative effects of these practices.

Mechanism	Management measures	Expected positive effects	Potential negative impacts
C input	Forestry: nonexporting harvest residues Grassland: choosing grazing over mowing Cropping: increasing soil OM input by: • Returning crop residues • Adding exogenous OM, incl. after transformation: e.g., organic waste products (Lashermes et al. 2009), biochars (Naisse et al. 2013; Hagemann et al. 2017) • Selection of plants with highly developed root systems or hyperexudative roots (Rasse et al. 2005) Associations for increasing C input diversity and quantity: • Rotations with permanent soil cover • Ley grassland • Agroforestry (Cardinael et al. 2017) • Silvo-pastoralism (Francaviglia et al. 2012)	Increase in C stock Addition of C mainly in the form of POM, no saturation issue POM input feeding of MAOM Improvement of soil physical and chemical fertility Improvement of soil life, better element cycling (Drinkwater and Snapp 2007) Transformed OM more persistent in soil (Möller 2015; Paolini et al. 2018)	Acceleration of OM turnover due to microbial growth and priming (Bernard et al. 2022; Perveen et al. 2019) POM sensitive to climate crisis events such as fire, warming Additional nutrient needs due to SOM stoichiometry constraints (Richardson et al. 2014) Addition of contaminants present in organic inputs Over-fertilization due to imbalance between the nutrient content of additional input and plant needs NH_3_, N_2_O emissions (Janz et al. 2021; Lashermes et al. 2021)
N input	Mineral N fertilization Introduction of N-fixing crops of the legume family N fertilization by human urine	Increased plant input to the soil due to increased primary production, Decrease in N-rich organic matter mining Supported production of N-rich microbial compounds, expected to persist in soil	Decreased C allocation to root inducing a decrease in soil C storage (Janssens et al. 2010) Nitrate and ammonia leaching and N_2_O emissions (Lemaire et al. 2021) Issue of social acceptance of urine fertilization (Martin et al. 2020)
Biology	Agro-ecological practices, such as non-tillage or no pesticide, implemented in the long-term favoring soil biotic legacy (Fanin and Bertrand 2016; Lu et al. 2018; Sauvadet et al. 2018),	Formation of biogenic structures enhancing SOM persistence (Lubbers et al. 2017) Better element cycling (Drinkwater and Snapp 2007) Enhancement of microbial compounds production, expected to persist in soil (Kallenbach et al. 2019)	Increased contribution of decomposer activity to CO_2_ production **(**Lubbers et al. 2013; Lejoly et al. 2021)
Spatial accessibility	Selection of plant species with deep root development favoring C input in the deep soil horizons, where biological activity is low (Rumpel and Kögel-Knabner 2011)	Increased C residence times compared to topsoil input (Balesdent et al. 2018)	Decrease in C storage due to stimulation of deep decomposer activity through fresh material supply (priming effect – Henneron et al. 2022)
	Liming	pH rise that facilitates OM/mineral interactions	
	Addition of mining or quarry wastes	Promotion of organomineral association formation	
	Reduced tillage or no-tillage (high controversy) (Dimassi et al. 2014)	Restriction of OM access to decomposers Enhanced substrate-decomposer contact at the macroscopic scale (mulch incorporation, grinding to reduce substrate size) (Angers et al. 1997) or at the microscopic scale (aggregate reorganization) (Six et al. 2000) Erosion control (Sun et al. 2015)	Accumulation of large amounts of plant residues at the soil surface where decomposer activity is most intense, instead of redistributing it over the soil profile Increase in N_2_O emissions (Guenet et al. 2021)

**Fig. 10:**
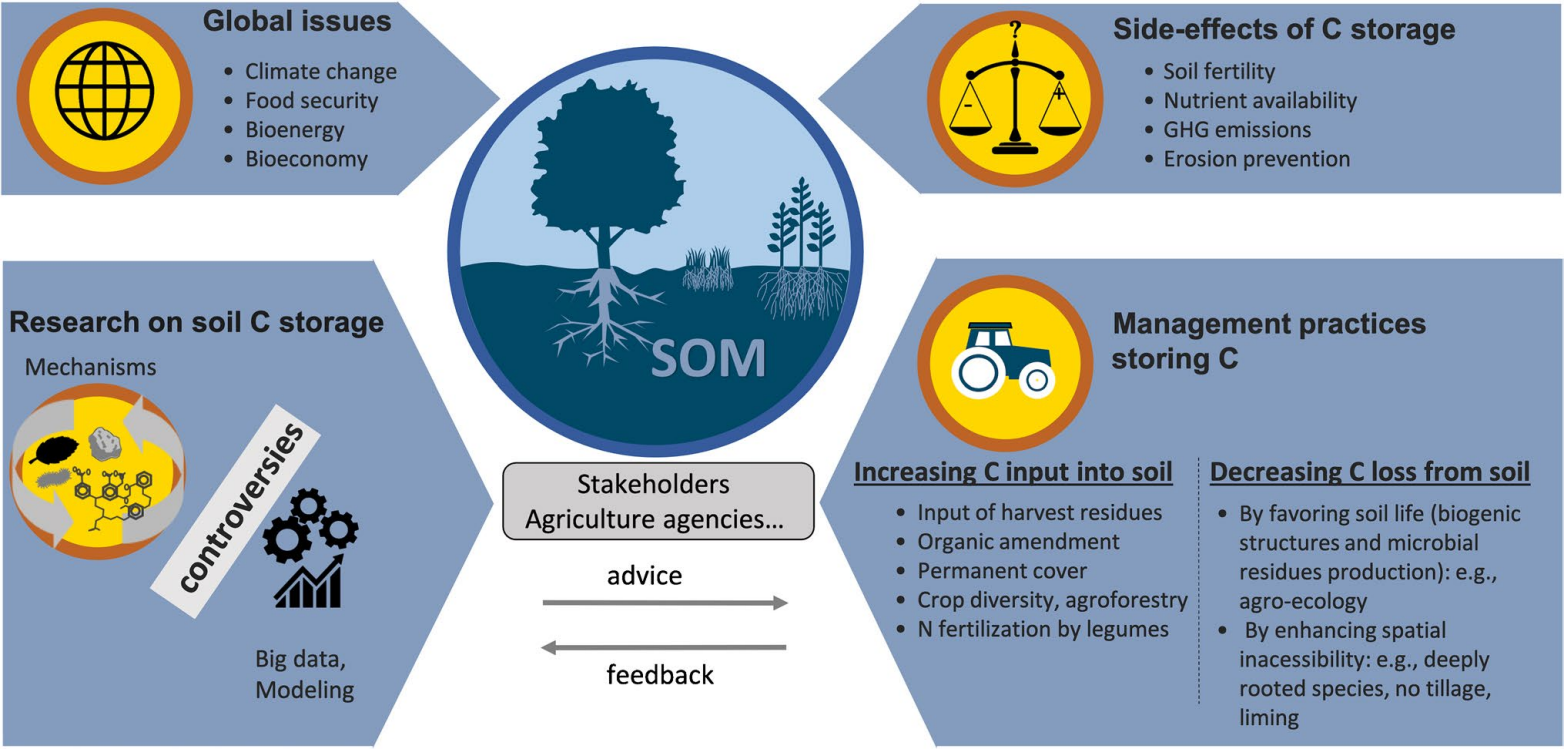
Synthesis of all the topics related to the increase of soil organic C storage that are addressed in this article. Management practices intended to promote C storage in a context of global constraints can be guided by results, theories, models, and debates arising from research, thanks to better communication between researchers and stakeholders. These practices aim to either increase C inputs or decrease C losses (see also Table 1).

The scientific community should make a particular effort to help quantify, with a site-specific approach, the possible adverse effects of the practices listed in Table 1, making use of long-term ecosystem research trials, database exploration, and modeling tools (see Section 3). Given the current challenges of climate change mitigation, food security, and bioeconomy growth, we consider that the practices favoring (i) soil life and the efficient nutrient cycling in the ecosystem and (ii) C input to the soil may be recommended more confidently than others (Fig. 10).

With respect to practices that favor soil life, the enhanced formation of biogenic structures protecting OM and the enhanced production of microbial compounds with a strong affinity for protecting mineral surfaces in soil (see Section 2.2) are expected to counterbalance the higher respiration also induced by an enhanced soil life (Liang et al. 2017, 2019) (Table 1). With respect to practices that enhance C input to soil, a recent point of contention is that soil C accumulation would require an additional immobilization of nutrients to meet the stoichiometric constraints of SOM. Given a C/N ratio of 12:15 in SOM, 100 Tg of additional N would be needed per year to reach the 4 per mil program objective (Schlesinger 2022; Soussana et al. 2019; van Groenigen et al. 2017). They could be provided by the application of fertilizers (about 210 tons of atmospheric N are currently fixed per year by the fertilizer industry—Fowler et al. 2013). But this measure would hardly be compatible with the agenda for C neutrality. Additional N needs could also be met through the adoption of agro-environmental practices limiting nutrient leaching in soil, preventing soil erosion, and/or enhancing N biological fixation (current estimates of annual biological fixation in terrestrial ecosystems range from 52 to 120 tons of N—Davies-Barnard and Friedlingstein 2020).

In any case, the implementation of practices favoring soil life and C input to the soil should be considered “win-win” strategies that promote the storage of C, nitrogen, and other nutrients in the form of OM and a better coupling of biogeochemical cycles (Drinkwater and Snapp 2007; Janzen 2005, 2006; Hufnagl-Eichiner et al. 2011). It is of utmost importance, though, that C storage in soil does not occur at the expense of nutrients provision to plants, fauna, and microorganisms.

## Conclusions and perspectives

By adopting an original approach based on the analysis of controversies on mechanisms of soil C storage, we demonstrate here that theories and concepts that appear at first glance to be antagonistic may in reality be quite complementary. Different theories can often be reconciled by considering variability in pedoclimatic properties, different decomposer functionalities, and changing environmental conditions. Future technical innovations will undoubtedly foster further debate and promote the development of novel emerging theories (Fig. 5).

Databases and models have an important role to play in the synthesis and dissemination of knowledge on mechanisms of SOM storage as well as in the translation of this knowledge into recommendations for practices beneficial to C storage. Nevertheless, the use of databases by the scientific community must be carried out cautiously, and the level of mechanistic description in models must be appropriate for the end user's goals and expectations.

While it is certainly true that much remains to be learned about mechanisms of C storage, their interactions, their relative importance in different environments, and how they can be integrated into databases and models, it is already possible to recommend and provide guidance on a certain number of management practices that have positive effects on soil C storage and for many ecosystem services (Fig. 10). When advising on beneficial practices, though, the scientific community must make it clear that there is no such thing as the absolute, definitive storage of C in soil (Dynarski et al. 2020). A given practice may lead to C storage for a certain period of time only. Moreover, this depends on the ecosystem’s characteristics and whether the practice can be maintained in the context of environmental, social or economic constraints. When choosing a practice, practitioners must be aware of the need to pursue it over the long term. The minimum storage timeframe considered in public policies, such as the Green Deal and C neutrality, is on the order of 20–30 years. The lack of quantitative knowledge on the impacts of recommended practices over time calls for long-term experiments over several decades in a wide variety of pedoclimatic situations, to simulate both the range of current conditions and those that we can expect with global warming, especially extreme climate events.

To conclude, it is critical that scientists are aware of existing debates in order to provide stakeholders with nuanced insights. They must ensure that a clear and consistent vocabulary is used and that the definitions and concepts that are debated within our community are explained and clarified for a wider audience, as done in this paper. Concurrently, stakeholders need to be aware of the need to interact with researchers as science evolves. To improve the translation of novel research findings into advice to policy-makers and practitioners, an improved dialogue between researchers and stakeholders (Fig. 10) is critical. This dialogue could be strengthened at all stages of research projects, from their construction—which could better involve stakeholders and more directly address questions from the field (Raous et al. 2020)—to the communication of research results in accessible media. For both communities, scientific networks that bring together researchers and stakeholders, such as CarboSMS in France, have an important role to play.

## Data Availability

Not applicable.

## Abbreviations

C: Carbon
MAOM: Mineral-associated organic matter
N: Nitrogen
OM: Organic matter
POM: Particulate organic matter
SOC: Soil organic carbon
SOM: Soil organic matter

## References

[CR1] Achat DL, Fortin M, Landmann G, Ringeval B, Augusto L (2015) Forest soil carbon is threatened by intensive biomass harvesting. Sci Rep-UK 5. 10.1038/srep15991. 10.1038/srep15991 PMC4632129 26530409

[CR2] Allison SD (2012). A trait-based approach for modelling microbial litter decomposition. Ecol Lett.

[CR3] Allory V, Séré G, Ouvrard S (2022) A meta-analysis of carbon content and stocks in Technosols and identification of the main governing factors. Eur J Soil Sci 73(1). 10.1111/ejss.13141

[CR4] Alvarez R, Berhongaray G (2021). Soil organic carbon sequestration potential of Pampean soils: comparing methods and estimation for surface and deep layers. Soil Res.

[CR5] Amelung W, Bossio D, de Vries W, Kögel-Knabner I, Lehmann J, Amundson R, Bol R, Collins C, Lal R, Leifeld J, Minasny B, Pan G, Paustian K, Rumpel C, Sanderman J, van Groenigen JW, Mooney S, van Wesemael B, Wander M, Chabbi A (2020). Towards a global-scale soil climate mitigation strategy. Nat Commun.

[CR6] Amelung W, Brodowski S, Sandhage-Hofmann A, Bol R (2008). Combining biomarker with stable isotope analyses for assessing the transformation and turnover of soil organic matter. Advances in Agronomy.

[CR7] Amin BAZ, Chabbert B, Moorhead D, Bertrand I (2014). Impact of fine litter chemistry on lignocellulolytic enzyme efficiency during decomposition of maize leaf and root in soil. Biogeochemistry.

[CR8] Angers DA, Recous S, Aita C (1997). Fate of carbon and nitrogen in water-stable aggregates during decomposition of (CN)-C-13-N-15-labelled wheat straw in situ. Eur J Soil Sci.

[CR9] Angst G, Mueller CW, Prater I, Angst Š, Frouz J, Jíková V, Peterse F, Nierop KGJ (2019). Earthworms act as biochemical reactors to convert labile plant compounds into stabilized soil microbial necromass. Commun Biol.

[CR10] Angst G, Mueller KE, Nierop K, Simpson MJ (2021). Plant- or microbial-derived? A review on the molecular composition of stabilized soil organic matter. Soil Biol Biochem.

[CR11] Antón SR, Derrien D, Urmeneta H, Van der Heijden G, Enrique A, Virto I (2022) Organic carbon storage and dynamics as affected by the adoption of irrigation in a cultivated calcareous mediterranean soil. Front Soil Sci 2(831775):1–18. 10.3389/fsoil.2022.831775

[CR12] Bailey VL, Pries CH, Lajtha K (2020). What do we know about soil carbon destabilization?. Environ Res Lett.

[CR13] Baldock JA, Skjemstad JO (2000). Role of the soil matrix and minerals in protecting natural organic materials against biological attack. Org Geochem.

[CR14] Balesdent J (1996). The significance of organic separates to carbon dynamics and its modelling in some cultivated soils. Eur J Soil Sci.

[CR15] Balesdent J, Basile-Doelsch I, Chadoeuf J, Cornu S, Derrien D, Fekiacova Z, Hatté C (2018). Atmosphere–soil carbon transfer as a function of soil depth. Nature.

[CR16] Bardgett RD, Van Der Putten WH (2014). Belowground biodiversity and ecosystem functioning. Nature.

[CR17] Barot S, Bornhofen S, Loeuille N, Perveen N, Shahzad T, Fontaine S (2014). Nutrient enrichment and local competition influence the evolution of plant mineralization strategy, a modelling approach. J Ecol.

[CR18] Barré P, Angers DA, Basile-Doelsch I, Bispo A, Cécillon L, Chenu C, Chevallier T, Derrien D, Eglin T, Pellerin S (2017) Ideas and perspectives: Can we use the soil carbon saturation deficit to quantitatively assess the soil carbon storage potential, or should we explore other strategies? Biogeosci Discuss 1-12. 10.5194/bg-2017-395

[CR19] Barré P, Plante AF, Cécillon L, Lutfalla S, Baudin F, Bernard S, Christensen BT, Eglin T, Fernandez JM, Houot S, Kätterer T, Le Guillou C, Macdonald A, van Oort F, Chenu C (2016). The energetic and chemical signatures of persistent soil organic matter. Biogeochemistry.

[CR20] Barré P, Quénéa K, Vidal A, Cécillon L, Christensen BT, Kätterer T, Macdonald A, Petit L, Plante AF, van Oort F, Chenu C (2018). Microbial and plant-derived compounds both contribute to persistent soil organic carbon in temperate soils. Biogeochemistry.

[CR21] Basile-Doelsch I, Balesdent J, Pellerin S (2020). Reviews and syntheses: The mechanisms underlying carbon storage in soil. Biogeosciences.

[CR22] Baveye PC, Schnee LS, Boivin P, Laba M, Radulovich R (2020). Soil organic matter research and climate change: merely re-storing carbon versus restoring soil functions. Front Environ Sci.

[CR23] Berg B (2014). Decomposition patterns for foliar litter—a theory for influencing factors. Soil Biol Biochem.

[CR24] Berhe AA, Harden JW, Torn MS, Kleber M, Burton SD, Harte J (2012) Persistence of soil organic matter in eroding versus depositional landform positions. J Geophys Res: Biogeosci 117(G2) 10.1029/2011JG001790

[CR25] Bernard L, Basile-Doelsch I, Derrien D, Fanin N, Fontaine S, Guenet B, Karimi B, Marsden C, Maron P-A (2022). Advancing the mechanistic understanding of the priming effect on soil organic matter mineralisation. Funct Ecol.

[CR26] Bertrand I, Chabbert B, Kurek B, Recous S (2006). Can the biochemical features and histology of wheat residues explain their decomposition in soil?. Plant Soil.

[CR27] Bertrand I, Viaud V, Daufresne T, Pellerin S, Recous S (2019). Stoichiometry constraints challenge the potential of agroecological practices for the soil C storage. A review. Agron Sustain Dev.

[CR28] Billings SA, Lajtha K, Malhotra A, Berhe AA, de Graaff MA, Earl S, Fraterrigo J, Georgiou K, Grandy S, Hobbie SE, Moore JAM, Nadelhoffer K, Pierson D, Rasmussen C, Silver WL, Sulman BN, Weintraub S, Wieder W (2021). Soil organic carbon is not just for soil scientists: measurement recommendations for diverse practitioners. Ecol Appl.

[CR29] Bird MI, Wynn JG, Saiz G, Wurster CM, McBeath A (2015). The pyrogenic carbon cycle. Annu Rev Earth Pl Sc.

[CR30] Bispo A, Andersen L, Angers DA, Bernoux M, Brossard M, Cécillon L, Comans RNJ, Harmsen J, Jonassen K, Lamé F, Lhuillery C, Maly S, Martin E, Mcelnea AE, Sakai H, Watabe Y, Eglin TK (2017). Accounting for carbon stocks in soils and measuring ghgs emission fluxes from soils: do we have the necessary standards?. Front Environ Sci.

[CR31] Blankinship JC, Berhe AA, Crow SE, Druhan JL, Heckman KA, Keiluweit M, Lawrence CR, Marín-Spiotta E, Plante AF, Rasmussen C, Schädel C (2018). Improving understanding of soil organic matter dynamics by triangulating theories, measurements, and models. Biogeochemistry.

[CR32] Bleuze L, Chabbert B, Lashermes G, Recous S (2020). Hemp harvest time impacts on the dynamics of microbial colonization and hemp stems degradation during dew retting. Ind Crop Prod.

[CR33] Boddy E, Hill PW, Farrah J, Jones DL (2007). Fast turnover of low molecular weight compounds of the dissolved organic carbon pool of temperate grassland field soils. Soil Biol Biochem.

[CR34] Bosatta E, Ågren GI (1999) Soil organic matter quality interpreted thermodynamically. Soil Biol Biochem 31:1889–1891. 10.1016/s0038-0717(99)00105-4

[CR35] Bousquet F, Le Page C (2004). Multi-agent simulations and ecosystem management: a review. Ecol Model.

[CR36] Boye K, Noël V, Tfaily MM, Bone SE, Williams KH, Bargar JR, Fendorf S (2017) Thermodynamically controlled preservation of organic carbon in floodplains. Nat Geosci 10(6):415–419. 10.1038/ngeo2940

[CR37] Bradford MA, Wieder WR, Bonan GB, Fierer N, Raymond PA, Crowther TW (2016). Managing uncertainty in soil carbon feedbacks to climate change. Nat Clim Chang.

[CR38] Budge K, Leifeld J, Hiltbrunner E, Fuhrer J (2011). Alpine grassland soils contain large proportion of labile carbon but indicate long turnover times. Biogeosciences.

[CR39] Cambou A, Shaw RK, Huot H, Vidal Beaudet L, Hunault G, Cannavo P, Nold F, Schwartz C (2018). Estimation of soil organic carbon stocks of two cities, New York City and Paris. Sci Total Environ.

[CR40] Cardinael R, Chevallier T, Cambou A, Béral C, Barthès GC, Dupraz C, Durand C, Kouakoua E, Chenu C (2017). Increased soil organic carbon stocks under agroforestry: A survey of six different sites in France. Agr Ecosyst Environt.

[CR41] Cécillon L, Barré P, Coissac E, Plante A, Rasse D (2015) Soil biogeochemistry in the age of big data. In: EGU general assembly conference abstracts, p 6964. https://ui.adsabs.harvard.edu/abs/2015EGUGA..17.6964C/abstract

[CR42] Cécillon L, Baudin F, Chenu C, Christensen BT, Franko U, Houot S, Kanari E, Katterer T, Merbach I, van Oort F, Poeplau C (2021) Partitioning soil organic carbon into its centennially stable and active fractions with machine-learning models based on Rock-Eval (R) thermal analysis (PARTY (SOC) v2. 0 and PARTY (SOC) v2. 0 (EU)). Geosci Model Dev 14:3879–3898. 10.5194/gmd-14-3879-2021

[CR43] Chabbi A, Lehmann J, Ciais P, Loescher HW, Cotrufo MF, Don A, San Clements M, Schipper L, Six J, Smith P, Rumpel C (2017). Aligning agriculture and climate policy. Nat Clim Chang.

[CR44] Chassé M, Luftalla S, Cécillon L, Baudin F, Abiven S, Chenu C, Barré P (2021) Long-term bare-fallow soil fractions reveal thermo-chemical properties controlling soil organic carbon dynamics. Biogeosciences 18:1703–1718. 10.5194/bg-18-1703-2021

[CR45] Chen S, Martin MP, Saby NP, Walter C, Angers DA, Arrouays D (2018). Fine resolution map of top-and subsoil carbon sequestration potential in France. Sci Total Environ.

[CR46] Chenu C, Angers DA, Barré P, Derrien D, Arrouays D, Balesdent J (2019). Increasing organic stocks in agricultural soils: Knowledge gaps and potential innovations. Soil Tillage Res.

[CR47] Chenu C, Stotzky G (2002) Interactions between microorganisms and soil particles: an overview. In: Interactions Between Soil Particles and Microorganisms—Impact on the Terrestrial Ecosystems. John Wiley and Sons, Chichester, pp 3–40

[CR48] Clemmensen KE, Bahr A, Ovaskainen O, Dahlberg A, Ekblad A, Wallander H, Stenlid J, Finlay RD, Wardle DA, Lindahl BD (2013). Roots and associated fungi drive long-term carbon sequestration in boreal forest. Science.

[CR49] Clivot H, Mouny J-C, Duparque A, Dinh J-L, Denoroy P, Houot S, Vertès F, Trochard R, Bouthier A, Sagot S, Mary B (2019). Modeling soil organic carbon evolution in long-term arable experiments with AMG model. Environ Model Softw.

[CR50] Coleman K, Jenkinson DS (1996). RothC-26.3-A Model for the turnover of carbon in soil. Evaluation of soil organic matter models.

[CR51] Cotrufo MF, Ranalli MG, Haddix ML, Six J, Lugato E (2019). Soil carbon storage informed by particulate and mineral-associated organic matter. Nat Geosci.

[CR52] Cotrufo MF, Soong JL, Horton AJ, Campbell EE, Haddix ML, Wall DH, Parton WJ (2015). Formation of soil organic matter via biochemical and physical pathways of litter mass loss. Nat Geosci.

[CR53] Craine JM, Morrow C, Fierer N (2007). Microbial nitrogen limitation increases decomposition. Ecology.

[CR54] Dangal SR, Sanderman J, Wills S, Ramirez-Lopez L (2019). Accurate and precise prediction of soil properties from a large mid-infrared spectral library. Soil Syst.

[CR55] Daufresne T, Loreau M (2001). Plant–herbivore interactions and ecological stoichiometry: when do herbivores determine plant nutrient limitation?. Ecol Lett.

[CR56] Davies-Barnard T, Friedlingstein P (2020). The global distribution of biological nitrogen fixation in terrestrial natural ecosystems. Glob Biogeochem Cycles.

[CR57] de Nobili M, Bravo C, Chen Y (2020). The spontaneous secondary synthesis of soil organic matter components: A critical examination of the soil continuum model theory. Appl Soil Ecol.

[CR58] Derrien D, Amelung W (2011). Computing the mean residence time of soil carbon fractions using stable isotopes: impacts of the model framework. Eur J Soil Sci.

[CR59] Derrien D, Marol C, Balabane M, Balesdent J (2006). The turnover of carbohydrate carbon in a cultivated soil estimated by ^13^C natural abundances. Eur J Soil Sci.

[CR60] Dignac MF, Bahri H, Rumpel C, Rasse DP, Bardoux G, Balesdent J, Girardin C, Chenu C, Mariotti A (2005). Carbon-13 natural abundance as a tool to study the dynamics of lignin monomers in soil: an appraisal at the Closeaux experimental field (France). Geoderma.

[CR61] Dignac MF, Derrien D, Barré P, Barot S, Cécillon L, Chenu C, Chevallier T, Freschet G, Garnier P, Guenet B, Hedde M, Klumpp K, Lashermes G, Maron PA, Nunan N, Roumet C, Basile-Doelsch I (2017) Increasing soil carbon storage: mechanisms, effects of agricultural practices and proxies. A review. Agron Sustain Dev 37:14. 10.1007/s13593-017-0421-2

[CR62] Dimassi B, Mary B, Wylleman R, Labreuche J, Couture D, Piraux F, Cohan JP (2014). Long-term effect of contrasted tillage and crop management on soil carbon dynamics during 41 years. Agric Ecosyst Environ.

[CR63] Don A, Rödenbeck C, Gleixner G (2013). Unexpected control of soil carbon turnover by soil carbon concentration. Environ Chem Lett.

[CR64] Drinkwater L, Snapp S (2007). Nutrients in agroecosystems: rethinking the management paradigm. Adv Agron.

[CR65] Dufour L, Herrmann A, Leloup J, Przybylski C, Foti L, Abbadie L, Nunan N (2021) Energetic return on investment determines overall soil microbial activity. Preprint 10.21203/rs.3.rs-388050/v1

[CR66] Dungait JAJ, Hopkins DW, Gregory AS, Whitmore AP (2012). Soil organic matter turnover is governed by accessibility not recalcitrance. Glob Chang Biol.

[CR67] Dunlop L, Veneu F (2019). Controversies in science. To teach or not to teach. Sci Educ.

[CR68] Durante S, Augusto L, Achat DL, Legout A, Brédoire F, Ranger J, Seynave I, Jabiol B, Pousse N (2019). Diagnosis of forest soil sensitivity to harvesting residues removal–A transfer study of soil science knowledge to forestry practitioners. Ecol Indic.

[CR69] Dynarski KA, Bossio DA, Scow KM (2020). Dynamic stability of soil C: Reassessing the "permanence" of soil carbon sequestration. Front Environ Sci.

[CR70] Ekschmitt K, Liu M, Vetter S, Fox O, Wolters V (2005). Strategies used by soil biota to overcome soil organic matter stability–why is dead organic matter left over in soil?. Geoderma.

[CR71] Erktan A, Rillig MC, Carminati A, Jousset A, Scheu S (2020). Protists and collembolans alter microbial community composition, C dynamics and soil aggregation in simplified consumer--prey systems. Biogeosciences.

[CR72] Fanin N, Bertrand I (2016). Aboveground litter quality is a better predictor than belowground microbial communities when estimating carbon mineralization along a land-use gradient. Soil Biol Biochem.

[CR73] Fanin N, Moorhead D, Bertrand I (2016). Eco-enzymatic stoichiometry and enzymatic vectors reveal differential C, N, P dynamics in decaying litter along a land-use gradient. Biogeochemistry.

[CR74] Farina R, Sándor R, Abdalla M (2021). Ensemble modelling, uncertainty and robust predictions of organic carbon in long-term bare-fallow soils. Glob Chang Biol.

[CR75] Favero A, Daigneault A, Sohngen B (2020). Forests: Carbon sequestration, biomass energy, or both?. Sci Adv.

[CR76] Finke P, Opolot E, Balesdent J, Berhe AA, Boeckx P, Cornu S, Harden J, Hatté C, Williams E, Doetterl S (2019). Can SOC modelling be improved by accounting for pedogenesis?. Geoderma.

[CR77] Floudas D, Binder M, Riley R, Barry K, Blanchette RA, Henrissat B, Martínez AT, Otillar R, Spatafora JW, Yadav JS, Aerts A (2012). The Paleozoic origin of enzymatic lignin decomposition reconstructed from 31 fungal genomes. Science.

[CR78] Fontaine S, Barot S, Barré P, Bdioui N, Mary B, Rumpel C (2007). Stability of organic carbon in deep soil layers controlled by fresh carbon supply. Nature Lett.

[CR79] Fontaine S, Henault C, Aamor A, Bdioui N, Bloor JMG, Maire V, Mary B, Revaillot S, Maron PA (2011). Fungi mediate long term sequestration of carbon and nitrogen in soil through their priming effect. Soil Biol Biochem.

[CR80] Fowler D, Coyle M, Skiba U, Sutton MA, Cape JN, Reis S, Sheppard LJ, Jenkins A, Grizzetti B, Galloway JN, Vitousek P, Leach A, Bouwman AF, Butterbach-Bahl K, Dentener F, Stevenson D, Amann M, Voss M (2013). The global nitrogen cycle in the twenty-first century. Philos Trans Royal Soc B: Biol Sci.

[CR81] Francaviglia R, Coleman K, Whitmore AP, Doro L, Urracci G, Rubino M, Ledda L (2012). Changes in soil organic carbon and climate change–Application of the RothC model in agro-silvo-pastoral Mediterranean systems. Agric Syst.

[CR82] Freschet GT, Violle C, Roumet C, Garnier E (2018) Interactions between soil and vegetation: structure of plant communities and soil functioning. In: Lemanceau P, Blouin M (ed) Component of the Critical Zone (Vol. 6: Ecology), ISTE Ltd and John Wiley & Sons, Inc, pp 83-104. 10.1002/9781119438274.ch5

[CR83] Garcet JP, Ordonez A, Roosen J, Vanclooster M (2006). Metamodelling: Theory, concepts and application to nitrate leaching modelling. Ecol Model.

[CR84] Georgiou K, Abramoff RZ, Harte J, Riley WJ, Torn MS (2017). Microbial community-level regulation explains soil carbon responses to long-term litter manipulations. Nat Commun.

[CR85] Georgiou K, Jackson RB, Vindušková O, Abramoff RZ, Ahlström A, Feng W, Harden JW, Pellegrini AA, Wayne Polley H, Soong JL, Riley WJ, Torn MS (2022). Global stocks and capacity of mineral-associated soil organic carbon. Nat Commun.

[CR86] Gleixner G (2013). Soil organic matter dynamics: a biological perspective derived from the use of compound-specific isotopes studies. Ecol Res.

[CR87] Gleixner G, Poirier N, Bol R, Balesdent J (2002). Molecular dynamics of organic matter in a cultivated soil. Org Geochem.

[CR88] Goodell B, Zhu Y, Kim S, Kafle K, Eastwood D, Daniel G, Jellison J, Yoshida M, Groom L, Pingali SV, O’Neill H (2017). Modification of the nanostructure of lignocellulose cell walls via a non-enzymatic lignocellulose deconstruction system in brown rot wood-decay fungi. Biotechnol Biofuels.

[CR89] Guenet B, Camino-Serrano M, Ciais P, Tifafi M, Maignan F, Soong JL, Janssens IA (2018). Impact of priming on global soil carbon stocks. Glob Chang Biol.

[CR90] Guenet B, Gabrielle B, Chenu C, Arrouays D, Balesdent J, Bernoux M, Bruni E, Caliman JP, Cardinael R, Chen S, Ciais P (2021). Can N_2_O emissions offset the benefits from soil organic carbon storage?. Glob Chang Biol.

[CR91] Hagedorn F, Bruderhofer N, Ferrari A, Niklaus PA (2015). Tracking litter-derived dissolved organic matter along a soil chronosequence using ^14^C imaging: biodegradation, physico-chemical retention or preferential flow?. Soil Biol Biochem.

[CR92] Hagedorn F, Gavazov K, Alexander JM (2019). Above and belowground linkages shape responses of mountain vegetation to climate change. Science.

[CR93] Hagemann N, Joseph S, Schmidt HP, Kammann CI, Harter J, Borch T, Young RB, Varga K, Taherymoosavi S, Elliott KW, McKenna A (2017). Organic coating on biochar explains its nutrient retention and stimulation of soil fertility. Nat Commun.

[CR94] Hall SJ, Ye C, Weintraub SR, Hockaday WC (2020). Molecular trade-offs in soil organic carbon composition at continental scale. Nat Geosci.

[CR95] Hassink J (1997). The capacity of soils to preserve organic C and N by their association with clay and silt particles. Plant Soil.

[CR96] Hatton PJ, Kleber M, Zeller B, Moni C, Plante AF, Townsend K, Gelhaye L, Lajtha K, Derrien D (2012). Transfer of litter-derived N to soil mineral–organic associations: evidence from decadal ^15^N tracer experiments. Org Geochem.

[CR97] Hemingway JD, Rothman DH, Grant KE, Rosengard SZ, Eglinton TI, Derry LA, Galy VV (2019). Mineral protection regulates long-term global preservation of natural organic carbon. Nature.

[CR98] Hemkemeyer M, Schwalb SA, Heinze S, Joergensen RG, Wichern F (2021). Functions of elements in soil microorganisms. Microbiol Res.

[CR99] Hénin S, Turc L (1949). Essai de fractionnement des matières organiques du sol. C Acad Agric Fr.

[CR100] Henneron L, Balesdent J, Alvarez G, Barré P, Baudin F, Basile-Doelsch I, Cécillon L, Fernandez-Martinez A, Hatté C, Fontaine S (2022). Bioenergetic control of soil carbon dynamics across depth. Nat Commun.

[CR101] Hicks LC, Lajtha K, Rousk J (2021). Nutrient limitation may induce microbial mining for resources from persistent soil organic matter. Ecology.

[CR102] Hill PW, Farrar JF, Jones DL (2008). Decoupling of microbial glucose uptake and mineralization in soil. Soil Biol Biochem.

[CR103] Hoffland E, Kuyper TW, Comans RNJ, Creamer RE (2020). Eco-functionality of organic matter in soils. Plant Soil.

[CR104] Hofman J, Dušek L (2003) Biochemical analysis of soil organic matter and microbial biomass composition—a pilot study. Eur J Soil Biol 39:217–224. 10.1016/J.EJSOBI.2003.08.002

[CR105] Hopkins DW, Dungait JAJ, Tilston EL (2010). Soil microbiology and nutrient cycling. Dixon, G. R..

[CR106] Hufnagl-Eichiner S, Wolf S, Drinkwater L (2011). Assessing social–ecological coupling: Agriculture and hypoxia in the Gulf of Mexico. Glob Environ Chang.

[CR107] Janssens I, Dieleman W, Luyssaert S, Subke J-A, Reichstein M, Ceulemans R, Ciais P, Dolman AJ, Grace J, Matteucci G, Papale D, Piao SL, Schulze ED, Tang J, Law BE (2010). Reduction of forest soil respiration in response to nitrogen deposition. Nat Geosci.

[CR108] Janusz G, Pawlik A, Sulej J, Świderska-Burek U, Jarosz-Wilkołazka A, Paszczyński A (2017). Lignin degradation: microorganisms, enzymes involved, genomes analysis and evolution. FEMS Microbiol Rev.

[CR109] Janz B, Havermann F, Lashermes G, Zuazo P, Engelsberger F, Torabi SM, Butterbach-Bahl K (2021) Effects of crop residue incorporation and properties on combined soil gaseous N_2_O, NO, and NH_3_ emissions—A laboratory-based measurement approach. Sci Total Environ 151051. 10.1016/j.scitotenv.2021.151051 10.1016/j.scitotenv.2021.151051 34710428

[CR110] Janzen HH (2005). Soil carbon: A measure of ecosystem response in a changing world?. Can J Soil Sci.

[CR111] Janzen HH (2006). The soil carbon dilemma: Shall we hoard it or use it?. Soil Biol Biochem.

[CR112] Jian J, Vargas R, Anderson-Teixeira KJ, Stell E, Herrmann V, Horn M, Kholod N, Manzon J, Marchesi R, Paredes D, Bond-Lamberty BP (2021). A Global Database of Soil Respiration Data, Version 5.0.

[CR113] Jouan J, De Graeuwe M, Carof M, Baccar R, Bareille N, Bastian S, Brogna D, Burgio G, Couvreur S, Cupiał M, Dumont B, Jacquot A-L, Magagnoli S, Makulska J, Maréchal K, Pérès G, Ridier A, Salou T, Tombarkiewicz B, Sgolastra F, Godinot O (2020). Learning Interdisciplinarity and Systems Approaches in Agroecology: Experience with the Serious Game SEGAE. Sustainability.

[CR114] Kallenbach CM, Frey SD, Grandy AS (2016). Direct evidence for microbial-derived soil organic matter formation and its ecophysiological controls. Nat Commun.

[CR115] Kallenbach CM, Grandy AS, Frey SD, Diefendorf AF (2015). Microbial physiology and necromass regulate agricultural soil carbon accumulation. Soil Biol Biochem.

[CR116] Kallenbach CM, Wallenstein MD, Schipanksi ME, Grandy AS (2019). Managing agroecosystems for soil microbial carbon use efficiency: ecological unknowns, potential outcomes, and a path forward. Front Microbiol.

[CR117] Keiluweit M, Bougoure J, Nico P, Pett-Ridge J, Weber PK, Kleber M (2015). Mineral protection of soil carbon counteracted by root exudates. Nat Clim Chang.

[CR118] Keiluweit M, Nico PS, Kleber M, Fendorf S (2016). Are oxygen limitations under recognized regulators of organic carbon turnover in upland soils?. Biogeochemistry.

[CR119] Keyvanshokouhi S, Cornu S, Lafolie F, Balesdent J, Guenet B, Moitrier N, Moitrier N, Nougier C, Finke P (2019). Effects of soil process formalisms and forcing factors on simulated organic carbon depth-distributions in soils. Sci Total Environ.

[CR120] Kirkby CA, Richardson AE, Wade LJ, Batten GD, Blanchard C, Kirkegaard JA (2013). Carbon-nutrient stoichiometry to increase soil carbon sequestration. Soil Biol Biochem.

[CR121] Kleber M (2010). What is recalcitrant soil organic matter?. Environ Chem.

[CR122] Kleber M, Bourg IC, Coward EK, Hansel CM, Myneni SC, Nunan N (2021). Dynamic interactions at the mineral–organic matter interface. Nature Rev Earth Environ.

[CR123] Kleber M, Eusterhues K, Keiluweit M, Mikutta C, Mikutta R, Nico PS (2015). Mineral–organic associations: formation, properties, and relevance in soil environments. AdvAgron.

[CR124] Kleber M, Johnson MG (2010). Advances in understanding the molecular structure of soil organic matter: implications for interactions in the environment. Adv Agron.

[CR125] Kleber M, Lehmann J (2019). Humic substances extracted by alkali are invalid proxies for the dynamics and functions of organic matter in terrestrial and aquatic ecosystems. J Environ Qual.

[CR126] Kleber M, Nico PS, Plante A, Filley T, Kramer M, Swanston C, Sollins P (2011). Old and stable soil organic matter is not necessarily chemically recalcitrant: implications for modeling concepts and temperature sensitivity. Glob Chang Biol.

[CR127] Kleerebezem R, Van Loosdrecht MC (2010). A generalized method for thermodynamic state analysis of environmental systems. Crit Rev Environ Sci Technol.

[CR128] Klotzbücher T, Kaiser K, Guggenberger G, Gatzek C, Kalbitz K (2011). A new conceptual model for the fate of lignin in decomposing plant litter. Ecology.

[CR129] Kögel-Knabner I (2002). The macromolecular organic composition of plant and microbial residues as inputs to soil organic matter. Soil Biol Biochem.

[CR130] Kögel-Knabner I, Amelung W (2021). Soil organic matter in major pedogenetic soil groups. Geoderma.

[CR131] Kögel-Knabner I, Guggenberger G, Kleber M, Kandeler E, Kalbitz K, Scheu S, Eusterhues K, Leinweber P (2008). Organo-mineral associations in temperate soils: Integrating biology, mineralogy, and organic matter chemistry. J Plant Nutr Soil Sci.

[CR132] Kohl L, Philben M, Edwards KA, Podrebarac FA, Warren J, Ziegler SE (2018). The origin of soil organic matter controls its composition and bioreactivity across a mesic boreal forest latitudinal gradient. Glob Chang Biol.

[CR133] Kopittke PM, Hernandez-Soriano MC, Dalal RC, Finn D, Menzies NW, Hoeschen C, Mueller CW (2018). Nitrogen-rich microbial products provide new organo-mineral associations for the stabilization of soil organic matter. Glob Chang Biol.

[CR134] Kravchenko AN, Guber AK (2017). Soil pores and their contributions to soil carbon processes. Geoderma.

[CR135] Lange M, Eisenhauer N, Sierra CA, Bessler H, Engels C, Griffiths RI, Mellado-Vázquez PG, Malik AA, Roy J, Scheu S, Steinbeiss S, Thomson BC, Trumbore SE, Gleixner G (2015). Plant diversity increases soil microbial activity and soil carbon storage. Nat Commun.

[CR136] LaRowe DE, Van Cappellen P (2011). Degradation of natural organic matter: a thermodynamic analysis. Geochim Cosmochim Acta.

[CR137] Larrère R (2018) Considérations sur les controverses. https://4p1000sete2018.sciencesconf.org/data/pages/Controverses_version_longue_RLarrere.pdf

[CR138] Lashermes G, Gainvors-Claisse A, Recous S, Bertrand I (2016) Enzymatic strategies and carbon use efficiency of a litter decomposing fungus grown on maize leaves, stems, and roots. Front Microbiol 10.3389/fmicb.2016.01315 10.3389/fmicb.2016.01315 PMC4999447 27617006

[CR139] Lashermes G, Moorhead D, Recous S, Bertrand I (2014) Interacting microbe and litter quality controls on litter decomposition: a modeling analysis. PLoS One 9.10.1371/journal.pone.0108769 10.1371/journal.pone.0108769 PMC4181322 25264895

[CR140] Lashermes G, Nicolardot B, Parnaudeau V, Thuries L, Chaussod R, Guillotin ML, Lineres M, Mary B, Metzger L, Morvan T, Tricaud A, Villette C, Houot S (2009). Indicator of potential residual carbon in soils after exogenous organic matter application. Eur J Soil Sci.

[CR141] Lashermes G, Recous S, Alavoine G, Janz B, Butterbach-Bahl K, Ernfors M, Laville P (2021) N_2_O emissions from decomposing crop residues are strongly linked to their initial soluble fraction and early C mineralization. Sci Total Environ 150883. 10.1016/j.scitotenv.2021.150883 10.1016/j.scitotenv.2021.150883 34653475

[CR142] Lavallée JM, Soong JL, Cotrufo MF (2020) Conceptualizing soil organic matter into particulate and mineral-associated forms to address global change in the 21st century. Glob Chang Biol 26(1):261–273. 10.1111/gcb.14859 10.1111/gcb.14859 31587451

[CR143] Lavelle P, Spain AV (2001). Soil Ecology.

[CR144] Lawrence CR, Beem-Miller J, Hoyt AM, Monroe G, Sierra CA, Stoner S, Heckman K, Blankinship JC, Crow SE, McNicol G, Trumbore S, Levine PA, Vindu O, Todd-Brown K, Rasmussen C, Hicks Pries CE, Schädel C, McFarlane K, Doetterl S, Hatté C, He Y, Treat C, Harden JW, Torn MS, Estop-Aragonés C, Asefaw Berhe A, Keiluweit M, Della Rosa Kuhnen A, Marin-Spiotta E, Plante AF, Thompson A, Shi Z, Schimel JP, Vaughn LJS, von Fromm SF, Wagai R (2020). An open-source database for the synthesis of soil radiocarbon data: International Soil Radiocarbon Database (ISRaD) version 1.0. Earth Syst Sci Data.

[CR145] Le Mer G, Barthod J, Dignac MF, Barré P, Baudin F, Rumpel C (2020). Inferring the impact of earthworms on the stability of organo-mineral associations, by Rock-Eval thermal analysis and ^13^C NMR spectroscopy. Org Geochem.

[CR146] Legout A, Hansson K, van der Heijden G, Laclau JP, Mareschal L, Nys C, Nicolas M, Saint-André L, Ranger J (2020). Chemical fertility of forest ecosystems. Part 2: Towards redefining the concept by untangling the role of the different components of biogeochemical cycling. For Ecol Managt.

[CR147] Lehmann J, Abiven S, Kleber M, Pan G, Singh BP, Sohi SP, Zimmerman AR (2015) Persistence of biochar in soil. In: Biochar for environmental management. Routledge, London, pp. 267–314

[CR148] Lehmann J, Hansel CM, Kaiser C, Kleber M, Maher K, Manzoni S, Nunan N, Reichstein M, Schimel JP, Torn MS, Wieder WR, Kögel-Knabner I (2020). Persistence of soil organic carbon caused by functional complexity. Nat Geosci.

[CR149] Lehmann J, Kleber M (2015). The contentious nature of soil organic matter. Nature.

[CR150] Lehmann J, Solomon D, Kinyangi J, Dathe L, Wirick S, Jacobsen C (2008). Spatial complexity of soil organic matter forms at nanometre scales. Nat Geosci.

[CR151] Leifeld J, Fuhrer J (2009). Long-term management effects on soil organic matter in two cold, high-elevation grasslands: clues from fractionation and radiocarbon dating. Eur J Soil Sci.

[CR152] Leifeld J, Kögel-Knabner I (2005). Soil organic matter fractions as early indicators for carbon stock changes under different land-use?. Geoderma.

[CR153] Leifeld J, Menichetti L (2018). The underappreciated potential of peatlands in global climate change mitigation strategies. Soil Biol Biochem.

[CR154] Leifeld J, von Lützow M (2014). Chemical and microbial activation energies of soil organic matter decomposition. Biol Fertil Soils.

[CR155] Lejoly J, Quideau S, Laganière J (2021). Invasive earthworms affect soil morphological features and carbon stocks in boreal forests. Geoderma.

[CR156] Lemaire G, Tang L, Béllanger G, Zhu Y, Jeuffroy MG (2021). Forward new paradigms for crop mineral nutrition and fertilization towards sustainable agriculture. Eur J Agron.

[CR157] Levavasseur F, Mary B, Christensen BT, Duparque A, Ferchaud F, Kätterer T, Lagrange H, Montenach D, Resseguier C, Houot S (2020). The simple AMG model accurately simulates organic carbon storage in soils after repeated application of exogenous organic matter. Nutr Cycl Agroecosyst.

[CR158] Liang C, Amelung W, Lehmann J, Kästner M (2019). Quantitative assessment of microbial necromass contribution to soil organic matter. Glob Chang Biol.

[CR159] Liang C, Schimel JP, Jastrow JD (2017). The importance of anabolism in microbial control over soil carbon storage. Nat Microbiol.

[CR160] Lorenz M, Hofmann D, Steffen B, Fischer K, Thiele-Bruhn S (2021) The molecular composition of extractable soil microbial compounds and their contribution to soil organic matter vary with soil depth and tree species. Sci Total Environ 146732. 10.1016/j.scitotenv.2021.146732

[CR161] Lu X, Lu X, Liao Y (2018) Effect of tillage treatment on the diversity of soil arbuscular mycorrhizal fungal and soil aggregate-associated carbon content. Front Microbiol 9.10.3389/fmicb.2018.02986 10.3389/fmicb.2018.02986 PMC6291503 30574132

[CR162] Lubbers IM, Pulleman MM, Van Groenigen JW (2017). Can earthworms simultaneously enhance decomposition and stabilization of plant residue carbon?. Soil Biol Biochem.

[CR163] Lubbers IM, van Groenigen KJ, Fonte SJ, Six J, Brussaard L, van Groenigen JW (2013). Greenhouse-gas emissions from soils increased by earthworms. Nat Clim Chang.

[CR164] Lugato E, Lavallee J, Haddix M, Panagos P, Cotrufo F (2021) Different climate sensitivity of particulate and mineral-associated organic matter. Nat Geosci 14. 10.1038/s41561-021-00744-x

[CR165] Malik AA, Roth VN, Hébert M, Tremblay L, Dittmar T, Gleixner G (2016). Linking molecular size, composition and carbon turnover of extractable soil microbial compounds. Soil Biol Biochem.

[CR166] Manzoni S, Taylor P, Richter A, Porporato A, Ågren GI (2012). Environmental and stoichiometric controls on microbial carbon-use efficiency in soils. New Phytol.

[CR167] Margida MG, Lashermes G, Moorhead DL (2020). Estimating relative cellulolytic and ligninolytic enzyme activities as functions of lignin and cellulose content in decomposing plant litter. Soil Biol Biochem.

[CR168] Marschner B, Brodowski S, Dreves A, Gleixner G, Gude A, Grootes PM, Hamer U, Heim A, Jandl G, Ji R, Kaiser K, Kalbitz K, Kramer C, Leinweber P, Rethemeyer J, Schäffer A, Schmidt MWI, Schwark L, Wiesenberg GLB (2008). How relevant is recalcitrance for the stabilization of organic matter in soils?. J Plant Nutr Soil Sci.

[CR169] Martin TMP, Esculier F, Levavasseur F, Houot S (2020) Human urine-based fertilizers: A review. Crit Rev Environ Sci Technol 10.1080/10643389.2020.1838214

[CR170] Miltner A, Bombach P, Schmidt-Brücken B, Kästner M (2012). SOM genesis: microbial biomass as a significant source. Biogeochemistry.

[CR171] Minasny B, Malone BP, McBratney AB, Angers DA, Arrouays D, Chambers A, Chaplot V, Chen ZS, Cheng K, Das BS, Field DJ, Gimona A, Hedley CB, Hong SY, Mandal B, Marchant BP, Martin M, McConkey BG, Mulder VL, O'Rourke S, Richer-de-Forges AC, Odeh I, Padarian J, Paustian K, Pan GX, Poggio L, Savin I, Stolbovoy V, Stockmann U, Sulaeman Y, Tsui CC, Vagen TG, van Wesemael B, Winowiecki L (2017). Soil carbon 4 per mille. Geoderma.

[CR172] Miyauchi S, Kiss E, Kuo A, Drula E, Kohler A, Sánchez-García M, Morin E, Andreopoulos B, Barry KW, Bonito G, Bué M (2020). Large-scale genome sequencing of mycorrhizal fungi provides insights into the early evolution of symbiotic traits. Nat Commun.

[CR173] Möller K (2015). Effects of anaerobic digestion on soil carbon and nitrogen turnover, N emissions, and soil biological activity. A review. Agron Sustain Dev.

[CR174] Monod J (1949). The growth of bacterial cultures. Annu Rev Microbiol.

[CR175] Moorhead DL, Lashermes G, Sinsabaugh RL (2012). A theoretical model of C- and N-acquiring exoenzyme activities, which balances microbial demands during decomposition. Soil Biol Biochem.

[CR176] Moorhead DL, Lashermes G, Sinsabaugh RL, Weintraub MN (2013). Calculating co-metabolic costs of lignin decay and their impacts on carbon use efficiency. Soil Biol Biochem.

[CR177] Myneni SCB (2019). Chemistry of natural organic matter—The next step: commentary on a humic substances debate. J Environ Qual.

[CR178] Naisse C, Alexis M, Plante A, Wiedner K, Glaser B, Pozzi A, Carcaillet C, Criscuoli I, Rumpel C (2013). Can biochar and hydrochar stability be assessed with chemical methods?. Org Geochem.

[CR179] Newcomb CJ, Qafoku NP, Grate JW, Bailey VL, De Yoreo JJ (2017). Developing a molecular picture of soil organic matter–mineral interactions by quantifying organo–mineral binding. Nat Commun.

[CR180] Olk DC, Bloom PR, Perdue EM, McKnight DM, Chen Y, Farenhorst A, Senesi N, Chin YP, Schmitt-Kopplin P, Hertkorn N, Harir M (2019). Environmental and agricultural relevance of humic fractions extracted by alkali from soils and natural waters. J Environ Qual.

[CR181] Paetsch L, Mueller CW, Rumpel C, Angst Š, Wiesheu AC, Girardin C, Ivleva NP, Niessner R, Kögel-Knabner I (2017). A multi-technique approach to assess the fate of biochar in soil and to quantify its effect on soil organic matter composition. Org Geochem.

[CR182] Panettieri M, Rumpel C, Dignac MF, Chabbi A (2017) Does grassland introduction into cropping cycles affect carbon dynamics through changes of allocation of soil organic matter within aggregate fractions? Sci Total Environ 576:251–263. 10.1016/j.scitotenv.2016.10.073 10.1016/j.scitotenv.2016.10.073 27788440

[CR183] Paolini V, Petracchini F, Segreto M, Tomassetti L, Naja N, Cecinato A (2018). Environmental impact of biogas: A short review of current knowledge. J Environ Sci Health Part A.

[CR184] Paul EA (2016). The nature and dynamics of soil organic matter: Plant inputs, microbial transformations, and organic matter stabilization. Soil Biol Biochem.

[CR185] Pellerin S, Bamiere L, Dimassi B, Launay C, Martin R, Schiavo M, Angers D, Augusto L, Balesdent J, Basile-Doelsch I, Bellassen V (2019) Storing carbon in French soils. Which potential regarding the 4 per 1000 objective, and to which cost? Synthesis of the study report, July 2019. https://www.inrae.fr/sites/default/files/pdf/etude-4-pour-1000-resume-en-francais-pdf-1_0.pdf

[CR186] Pengerud A, Dignac MF, Certini G, Strand LT, Forte C, Rasse DP (2017). Soil organic matter molecular composition and state of decomposition in three locations of the European Arctic. Biogeochemistry.

[CR187] Perveen N, Barot S, Alvarez G, Klumpp K, Martin R, Rapaport A, Herfurth D, Louault F, Fontaine S (2014). Priming effect and microbial diversity in ecosystem functioning and response to global change: a modeling approach using the SYMPHONY model. Glob Chang Biol.

[CR188] Perveen N, Barot S, Maire V, Cotrufo MF, Shahzad T, Blagodatskaya E, Stewart CE, Ding W, Siddiq MR, Dimassi B, Mary B, Fontaine S (2019). Universality of priming effect: An analysis using thirty five soils with contrasted properties sampled from five continents. Soil Biol Biochem.

[CR189] Pinheiro M, Garnier P, Beguet J, Martin Laurent F, Vieuble Gonod L (2015). The millimetre-scale distribution of 2,4-D and its degraders drives the fate of 2,4-D at the soil core scale. Soil Biol Biochem.

[CR190] Plante A, Fernández JM, Haddix ML, Steinweg JM, Conant RT (2011). Biological, chemical and thermal indices of soil organic matter stability in four grassland soils. Soil Biol Biochem.

[CR191] Plante A, Beaupré S, Roberts M, Baisden T (2013). Distribution of radiocarbon ages in soil organic matter by thermal fractionation. Radiocarbon.

[CR192] Poeplau C, Don A (2013). Sensitivity of soil organic carbon stocks and fractions to different land-use changes across Europe. Geoderma.

[CR193] Poeplau C, Don A, Six J, Kaiser M, Benbi D, Chenu C, Cotrufo MF, Derrien D, Gioacchini P, Grand S, Gregorich E, Griepentrog M, Gunina A, Haddix M, Kuzyakov Y, Kühnel A, Macdonald LM, Soong J, Trigalet S, Vermeire ML, Rovira P, van Wesemael B, Wiesmeier M, Yeasmin S, Yevdokimov I, Nieder R (2018). Isolating organic carbon fractions with varying turnover rates in temperate agricultural soils – A comprehensive method comparison. Soil Biol Biochem.

[CR194] Poirier V, Roumet C, Munson AD (2018). The root of the matter: linking root traits and soil organic matter stabilization processes. Soil Biol Biochem.

[CR195] Puissant J, Mills RTE, Robroek BJM, Gavazov K, Perrette Y, De Danieli S, Spiegelberger T, Buttler A, Brun JJ, Cécillon L (2017). Climate change effects on the stability and chemistry of soil organic carbon pools in a subalpine grassland. Biogeochemistry.

[CR196] Raous S, King C, Alletto L, Bougon N, Chenu C, Cortet J, Derrien D, Dictor MC, François Y, Keller C, Perrin AS, Pousse N, Rémy E, Rennes S, Servain F, Tournebize J (2020) Recommandations méthodologiques pour le montage de projets collaboratifs entre acteurs de la Recherche et des Territoires. Rapport du Comité Scientifique Technique et d'Innovation (CSTI) du Réseau National d'Expertise Scientifique et Technique sur les sols (RNEST). 10.13140/RG.2.2.35757.82405

[CR197] Rasse DP, Rumpel C, Dignac MF (2005). Is soil carbon mostly root carbon? Mechanisms for a specific stabilisation. Plant Soil.

[CR198] Recous S, Lashermes G, Bertrand I, Duru M, Pellerin S (2019) C–N–P decoupling processes linked to arable cropping management systems in relation with intensification of production. In: Agroecosystem Diversity: Reconciling contemporary agriculture and environmental quality. Academic Press. Elsevier, London, pp 35–53. 10.1016/B978-0-12-811050-8.00003-0/B978-0-12-811050-8.00003-0

[CR199] Rees F, Dagois R, Derrien D, Fiorelli JL, Watteau F, Morel JL, Schwartz C, Simonnot MO, Séré G (2019). Storage of carbon in constructed technosols: in situ monitoring over a decade. Geoderma.

[CR200] Richardson AE, Kirkby CA, Banerjee S, Kirkegaard JA (2014). The inorganic nutrient cost of building soil carbon. Carbon Manag.

[CR201] Rillig MC (2004). Arbuscular mycorrhizae, glomalin, and soil aggregation. Can J Soil Sci.

[CR202] Rocci KS, Lavallee JM, Stewart CE, Cotrufo MF (2021). Soil organic carbon response to global environmental change depends on its distribution between mineral-associated and particulate organic matter: A meta-analysis. Sci Total Environ.

[CR203] Rovira P, Kurz-Besson C, Coûteaux MM, Vallejo VR (2008). Changes in litter properties during decomposition: a study by differential thermogravimetry and scanning calorimetry. Soil Biol Biochem.

[CR204] Rowley MC, Grand S, Verrecchia EP (2018). Calcium-mediated stabilization of soil organic carbon. Biogeochemistry.

[CR205] Rumpel C, Kögel-Knabner I (2011). Deep soil organic matter—a key but poorly understood component of terrestrial C cycle. Plant Soil.

[CR206] Saadat NP, Nies T, Rousset Y, Ebenhöh O (2020). Thermodynamic Limits and Optimality of Microbial Growth. Entropy.

[CR207] Sainte-Marie J, Barrandon M, Saint-André L, Gelhaye E, Martin F, Derrien D (2021). C-STABILITY an innovative modeling framework to leverage the continuous representation of organic matter. Nat Commun.

[CR208] Sanderman J, Hengl T, Fiske GJ (2017). Soil carbon debt of 12,000 years of human land use. P Natl Acad Sci USA.

[CR209] Sathre R, O’Connor J (2010). Meta-analysis of greenhouse gas displacement factors of wood product substitution. Environ Sci Pol.

[CR210] Sauvadet M, Lashermes G, Alavoine G, Recous S, Chauvat M, Maron PA, Bertrand I (2018). High carbon use efficiency and low priming effect promote soil C stabilization under reduced tillage. Soil Biol Biochem.

[CR211] Schimel JP, Schaeffer SM (2012) Microbial control over carbon cycling in soil. Front Microbiol 10.3389/fmicb.2012.00348 10.3389/fmicb.2012.00348 PMC3458434 23055998

[CR212] Schlesinger WH (2022) Biogeochemical constraints on climate change mitigation through regenerative farming. Biogeochemistry, 1-9. 10.1007/s10533-022-00942-8

[CR213] Schmidt MWI, Torn MS, Abiven S, Dittmar T, Guggenberger G, Janssens IA, Kleber M, Kögel-Knabner I, Lehmann J, Manning DAC, Nannipieri P, Rasse DP, Weiner S, Trumbore SE (2011). Persistence of soil organic matter as an ecosystem property. Nature.

[CR214] Schneider T, Keiblinger K, Schmid E, Sterflinger-Gleixner K, Ellersdorfer G, Roschitzki B, Richter A, Eberl L, Zechmeister-Boltenstern S, Riedel K (2012). Who is who in litter decomposition? Metaproteomics reveals major microbial players and their biogeochemical functions. ISME J.

[CR215] Schrumpf M, Kaiser K, Mayer A, Hempel G, Trumbore S (2021). Age distribution, extractability, and stability of mineral-bound organic carbon in central European soils. Biogeosciences.

[CR216] Schulten HR, Leinweber P (1996). Characterization of humic and soil particles by analytical pyrolysis and computer modeling. J Anal Appl Pyrolysis.

[CR217] Schuur EAG, Bockheim J, Canadell JG, Euskirchen E, Field CB, Goryachkin SV, Hagemann S, Kuhry P, Lafleur PM, Lee H, Mazhitova G, Nelson FE, Rinke A, Romanovsky VE, Shiklomanov N, Tarnocai C, Venevsky S, Vogel JG, Zimov SA (2008). Vulnerability of permafrost carbon to climate change: implications for the global. Bio Sci.

[CR218] Shahzad T, Chenu C, Genet P, Barot S, Perveen N, Mougin C, Fontaine S (2015). Contribution of exudates, arbuscular mycorrhizal fungi and litter depositions to the rhizosphere priming effect induced by grassland species. Soil Biol Biochem.

[CR219] Shi Z, Crowell S, Luo Y, Moore B (2018). Model structures amplify uncertainty in predicted soil carbon responses to climate change. Nat Commun.

[CR220] Six J, Elliott E, Paustian K (1999). Aggregate and soil organic matter dynamics under conventional and no-tillage systems. Soil Sci Soc Am J.

[CR221] Six J, Elliott ET, Paustian K (2000). Soil macroaggregate turnover and microaggregate formation: a mechanism for C sequestration under no-tillage agriculture. Soil Biol Biochem.

[CR222] Six J, Frey SD, Thiet RK, Batten KM (2006). Bacterial and fungal contributions to carbon sequestration in agroecosystems. Soil Sci Soc Am J.

[CR223] Sollins P, Homann P, Caldwell BA (1996). Stabilization and destabilization of soil organic matter: mechanisms and controls. Geoderma.

[CR224] Soucémarianadin L, Cécillon L, Chenu C, Baudin F, Nicolas M, Girardin C, Delahaie A, Barré P (2019). Heterogeneity of the chemical composition and thermal stability of particulate organic matter in French forest soils. Geoderma.

[CR225] Soussana JF, Lutfalla S, Ehrhardt F, Rosenstock T, Lamanna C, Havlík P, Richards M, Wollenberg EL, Chotte JL, Torquebiau E, Ciais P, Smith P, Lal R (2019). Matching policy and science: Rationale for the 4 per 1000-soils for food security and climate initiative. Soil Tillage Res.

[CR226] Sposito G (2008) The chemistry of soils. Oxford University Press, New York

[CR227] Stevenson FJ (1994). Humus chemistry: genesis, composition, reactions.

[CR228] Sulman BN, Brzostek ER, Medici C, Shevliakova E, Menge DNL, Phillips RP (2017). Feedbacks between plant N demand and rhizosphere priming depend on type of mycorrhizal association. Ecol Lett.

[CR229] Sulman BN, Moore JA, Abramoff R, Averill C, Kivlin S, Georgiou K, Sridhar B, Hartman MD, Wang G, Wieder WR, Bradford MA, Luo Y, Mayes MA, Morrison E, Riley WE, Salazar A, Schimel JP, Tang J, Classen AT (2018). Multiple models and experiments underscore large uncertainty in soil carbon dynamics. Biogeochemistry.

[CR230] Sun Y, Zeng Y, Shi Q, Pan X, Huang S (2015). No-tillage controls on runoff: A meta-analysis. Soil Tillage Res.

[CR231] Sutton R, Sposito G (2005). Molecular structure in soil humic substances: the new view. Environ Sci Technol.

[CR232] The Future of Food and Agriculture—Trends and Challenges (2017) FAO, Rome, Italie. http://www.fao.org/3/a-i6583e.pdf

[CR233] Tivet F, de Moraes C, Sá BPR, Letourmy P, Briedis C, Ferreira AO, dos Santos B, Thiago Massao Inagaki J (2012). Soil carbon inventory by wet oxidation and dry combustion methods: Effects of land use, soil texture gradients, and sampling septh on the linear model of C-equivalent correction factor. Soil Sci Soc Am J.

[CR234] Torn MS, Vitousek PM, Trumbore SE (2005). The influence of nutrient availability on soil organic matter turnover estimated by incubations and radiocarbon modeling. Ecosystems.

[CR235] Traore O, Groleau-Renaud V, Plantureux S, Tubeileh A, Boeuf-Tremblay V (2000). Effect of root mucilage and modelled root exudates on soil structure. Eur J Soil Sci.

[CR236] Trumbore S (2009). Radiocarbon and soil carbon dynamics. Annu Rev Earth Planet Sci.

[CR237] Uroz S, Calvaruso C, Turpault MP, Frey-Klett P (2009). Mineral weathering by bacteria: ecology, actors and mechanisms. Trends Microbiol.

[CR238] Uroz S, Kelly LC, Turpaul MP, Lepleux C, Frey-Klett P (2015). The mineralosphere concept: mineralogical control of the distribution and function of mineral-associated bacterial communities. Trends Microbiol.

[CR239] Uroz S, Picard L, Turpault MP, Auer L, Armengaud J, Oger P (2020). Dual transcriptomics and proteomics analyses of the early stage of interaction between Caballeronia mineralivorans PML1(12) and mineral. Environ Microbiol.

[CR240] Valade A, Luyssaert S, Vallet P, Djomo SN, Van Der Kellen IJ, Bellassen V (2018) Carbon costs and benefits of France's biomass energy production target. CBM 10.1186/s13021-018-0113-5 10.1186/s13021-018-0113-5 PMC6292836 30547241

[CR241] van Groenigen JW, van Kessel C, Hungate BA, Oenema O, Powlson DS, van Groenigen KJ (2017) Sequestering Soil Organic Carbon: A Nitrogen Dilemma Environ. Sci Technol 51:4738–4739. 10.1021/acs.est.7b01427 10.1021/acs.est.7b01427 28426199

[CR242] Vestergaard G, Schulz S, Schöler A, Schloter M (2017). Making big data smart—how to use metagenomics to understand soil quality. Biol Fertil Soils.

[CR243] Vidal A, Watteau F, Remusat L, Mueller CW, Nguyen Tu TT, Buegger F, Derenne S, Quenea K (2019). Earthworm cast formation and development: a shift from plant litter to mineral associated organic matter. Front Environ Sci.

[CR244] Virto I, Moni C, Swanston C, Chenu C (2010). Turnover of intra- and extra-aggregate organic matter at the silt-size scale. Geoderma.

[CR245] Viscarra Rossel RA, Lee J, Behrens T, Luo Z, Baldock J, Richards A (2019). Continental-scale soil carbon composition and vulnerability modulated by regional environmental controls. Nat Geosci.

[CR246] von Lützow M, Kögel-Knabner I, Ekschmitt K, Flessa H, Guggenberger G, Matzner E, Marschner B (2007). SOM fractionation methods: relevance to functional pools and to stabilization mechanisms. Soil Biol Biochem.

[CR247] Wadoux AC, Samuel-Rosa A, Poggio L, Mulder VL (2020). A note on knowledge discovery and machine learning in digital soil mapping. Eur J Soil Sci.

[CR248] Wander MM (2004). Soil organic matter fractions and their relevance to soil function. Soil organic matter in sustainable agriculture.

[CR249] Wang C, Qu L, Yang L, Liu D, Morrissey E, Miao R, Liu Z, Wang Q, Fang Y, Bai E (2021). Large-scale importance of microbial carbon use efficiency and necromass to soil organic carbon. Glob Chang Biol.

[CR250] Wang T, Tian Z, Bengtson P, Tunlid A, Persson P (2017). Mineral surface-reactive metabolites secreted during fungal decomposition contribute to the formation of soil organic matter. Environ Microbiol.

[CR251] Waring BG, Sulman BN, Reed S, Smith AP, Averill C, Creamer CA, Cusack DF, Hall SJ, Jastrow JD, Jilling A, Kemner KM KM, Kleber M, Xiao LXJA, Pett-Ridge J, Schulz M (2020). From pools to flow: The PROMISE framework for new insights on soil carbon cycling in a changing world. Glob Chang Biol.

[CR252] Wieder WR, Hartman MD, Sulman BN, Wang YP, Koven CD, Bonan GB (2018). Carbon cycle confidence and uncertainty: Exploring variation among soil biogeochemical models. Glob Chang Biol.

[CR253] Williams EK, Plante AF (2018). A bioenergetic framework for assessing soil organic matter persistence. Front Earth Sci.

[CR254] Witzgall K, Vidal A, Schubert DI, Höschen C, Schweizer SA, Buegger F, Pouteau V, Chenu C, Mueller CW (2021). Particulate organic matter as a functional soil component for persistent soil organic carbon. Nat Commun.

[CR255] Yu GH, Kuzyakov Y (2021). Fenton chemistry and reactive oxygen species in soil: Abiotic mechanisms of biotic processes, controls and consequences for carbon and nutrient cycling. Earth Sci Rev.

[CR256] Zechmeister-Boltenstern S, Keiblinger KM, Mooshammer M, Penuelas J, Richter A, Sardans J, Wanek W (2015). The application of ecological stoichiometry to plant-microbial-soil organic matter transformations. Ecol Monogr.

[CR257] Zhang J, Presley GN, Hammel KE, Ryu JS, Menke JR, Figueroa M, Hu D, Orr G, Schilling JS (2016). Localizing gene regulation reveals a staggered wood decay mechanism for the brown rot fungus Postia placenta. P Natl Acad Sci USA.

